# Annexin A2 is a novel Cellular Redox Regulatory Protein involved in Tumorigenesis

**DOI:** 10.18632/oncotarget.375

**Published:** 2011-12-20

**Authors:** Patricia Alexandra Madureira, Richard Hill, Victoria Ann Miller, Carman Giacomantonio, Patrick Wing Kwong Lee, David Morton Waisman

**Affiliations:** ^1^ Departments of Biochemistry & Molecular Biology and Pathology; ^2^ Department of Microbiology and Immunology; ^3^ Department of Medicine, Dalhousie University, Halifax, Nova Scotia, B3H 4R2, Canada

**Keywords:** ANXA2, reactive oxygen species (ROS), redox regulation, oxidative stress, tumorigenesis

## Abstract

Annexins are a structurally related family of calcium and phospholipid-binding proteins that are involved in the regulation of a wide range of molecular and cellular processes. Annexin A2 is unique among the annexins in that it possesses redox sensitive cysteine(s). The ubiquitous and abundant expression of ANXA2 in cells and its reactivity with hydrogen peroxide led us to hypothesize that this protein could play a role in cellular redox regulation. Here we show that ANXA2 protein levels are induced by hydrogen peroxide. Furthermore, depletion of ANXA2 resulted in the elevation of cellular reactive oxygen species (ROS) upon oxidative stress, increased activation of the ROS-induced pro-apoptotic kinases, JNK, p38 and Akt and elevated sensitivity to ROS-mediated cellular damage/death. ANXA2-null mice showed significantly elevated protein oxidation in the liver and lung tissues compared to WT mice. ANXA2 depleted cancer cells showed enhanced cellular protein oxidationconcomitant with decreased tumor growth compared to control cancer cells andboth the oxidation of cellular proteins and tumor growth deficit werereversed by the antioxidant N-acetyl cysteine, indicating that ANXA2 plays akey role in the regulation of cellular redox during tumorigenesis. *Ex-vivo* human cancer studies showed that up-regulation of the reduced form of ANXA2 is associated with protection of the tumor proteins from oxidation. In summary, our results indicate that ANXA2 plays an important role incellular redox regulation by protecting cells from oxidative stress, aneffect that is particularly important during tumorigenesis.

## INTRODUCTION

Reactive oxygen species (ROS) are oxygen-containing reactive chemical species which include such biologically important molecules as superoxide, nitric oxide, hydroxyl radical and hydrogen peroxide (H_2_O_2_). Endogenous H_2_O_2_ is a by-product of mitochondrial respiration [1]. In addition, various signaling molecules, including growth factors, cytokines, hormones and neurotransmitters induce increases in intracellular H_2_O_2_ through the activation of NADPH oxidases (Nox). H_2_O_2_-dependent signaling has been implicated in diverse processes such as regulation of cell proliferation, differentiation, migration and apoptosis [2, 3, 4]. The production of a cytotoxic molecule has obvious potential risks to the cells as H_2_O_2_ is a major contributor to DNA damage, protein oxidation and lipid peroxidation [5, 6]. Cells therefore show a biphasic response to increased H_2_O_2_ levels; this is due to its role as a second messenger in intracellular signaling cascades (proliferative effect) and at high concentrations as an oxidant of proteins, lipids and nucleic acids (apoptotic and/or necrotic effect).

Cancer cells typically express higher levels of ROS compared to normal cells which gives them both a proliferative advantage and also promotes malignant progression [7, 8]. Ionizing radiation (IR) and chemotherapeutic drugs that elevate ROS levels are toxic to cancer cells because the same dosage of drug or radiation will cause death of the cancer cell due to ROS overload, but will be less toxic to neighboring normal cells which contain lower endogenous levels of ROS.

Redox-sensitive cysteines are the primary target for protein oxidation by H_2_O_2_. The amino acid cysteine can exist in proteins as a Cys sulfhydryl group (Cys-SH) or a Cys thiolate anion group (Cys-S^−^). Since the pKa value of most protein Cys residues is about 8.5, at neutral pH most Cys residues are in the Cys-SH form. However, a very small subset of proteins contain Cys residue(s) in the Cys-S- form at neutral pH typically due to the stabilization of this form by salt bridges to positively charged amino acids. Only the Cys-S^−^ form is readily susceptible to oxidation by ROS and for this reason is referred to as a redox-sensitive cysteine. The redox-sensitive cysteines function as sensors of H_2_O_2_ and the oxidation of these residues affects the biological activity of the protein. For example, certain transcription factors such as JunD have a redox sensitive cysteine strategically positioned within their DNA binding domains such that oxidation of this cysteine by H_2_O_2_ blocks DNA-binding activity. Alternatively, antioxidant proteins utilize their redox sensitive cysteine(s) catalytically to reduce H_2_O_2_ to H_2_O.

Cells have developed several antioxidant systems to avoid ROS induced damage. These include catalase, superoxide dismutase, glutathione peroxidase and thioredoxin peroxidase (peroxiredoxins). The primary redox sensitive cysteine reductases are thioredoxin (Trx) and glutathione (GSH). Trx regulates signaling molecules, transcription factors, peroxidases and mediates redox-regulated gene expression [9]. During reduction of target proteins, Trx is oxidized and subsequently reduced/regenerated by thioredoxin reductase and NADPH. The Trx, Trx reductase and NADPH, collectively called the Trx redox system, constitute a fundamental antioxidant system.

Annexins are a structurally related family of calcium and phospholipid-binding proteins that are involved in the regulation of a range of molecular and cellular processes [10, 11]. The de-regulated expression of annexins has been shown to constitute a valuable marker of cancer progression [12, 13]. Annexin A2 (ANXA2) in particular has been positively associated with malignant progression [14] and resistance to chemotherapy [15, 16]. ANXA2 is unique among the annexins in that it possesses redox sensitive cysteine(s). Our laboratory has shown that extracellular ANXA2 binds to and reduces the enzyme plasmin and that ANXA2 oxidized during this reaction is subsequently reduced by the thioredoxin (Trx) redox system [17]. The presence of redox sensitive cysteine(s) on ANXA2 that can participate in cycles of oxidation and reduction, coupled with its ubiquitous and abundant expression in cells led us to hypothesize that this protein could play a role in cellular redox regulation.

Here, we report a novel role for ANXA2 as a redox regulatory protein. We show that upon oxidative stress, ANXA2 depleted cells display higher levels of ROS, increased activation of the ROS-induced pro-apoptotic kinases: p38, JNK and Akt and higher sensitivity to oxidative stress-mediated cell death compared to control cells. We further identify the Cys^8^ residue as the redox sensitive cysteine of ANXA2 and show that this cysteine is reversibly oxidized by H_2_O_2_ and reduced by the thioredoxin redox system. *In vivo* studies show a significant increase in protein oxidation in the liver and lung tissues of ANXA2-null mice compared to WT mice. Furthermore, the growth of tumors resulting from the subcutaneous injection of ANXA2-depleted human cancer cell lines, HT1080 and A549, in mice showed severe growth impairment compared to control cells. Intraperitoneal injection of the antioxidant, N-acetyl cysteine (NAC) enabled these tumors to grow at a similar rate as the control tumors. We also observed enhanced protein oxidation in the ANXA2 depleted HT1080 tumors compared to control tumors, which was prevented by NAC treatment. These results show that replacement of ANXA2 by another antioxidant, such as NAC, reverses the tumor growth deficit phenotype observed in the ANXA2 depleted cells, indicating that ANXA2 is a redox regulatory protein that plays a key role in tumorigenesis. *Ex-vivo* human cancer studies showed that in general, cancer cells express significantly higher levels of the reduced form of ANXA2 compared to normal tissue and that the up-regulation of the levels of reduced ANXA2 correlate with protection from oxidation of the proteins in these tumors, indicating that ANXA2 may function as a redox regulatory protein in human tumors.

## RESULTS

### ANXA2 protein is a redox sensor

**Figure 1:**
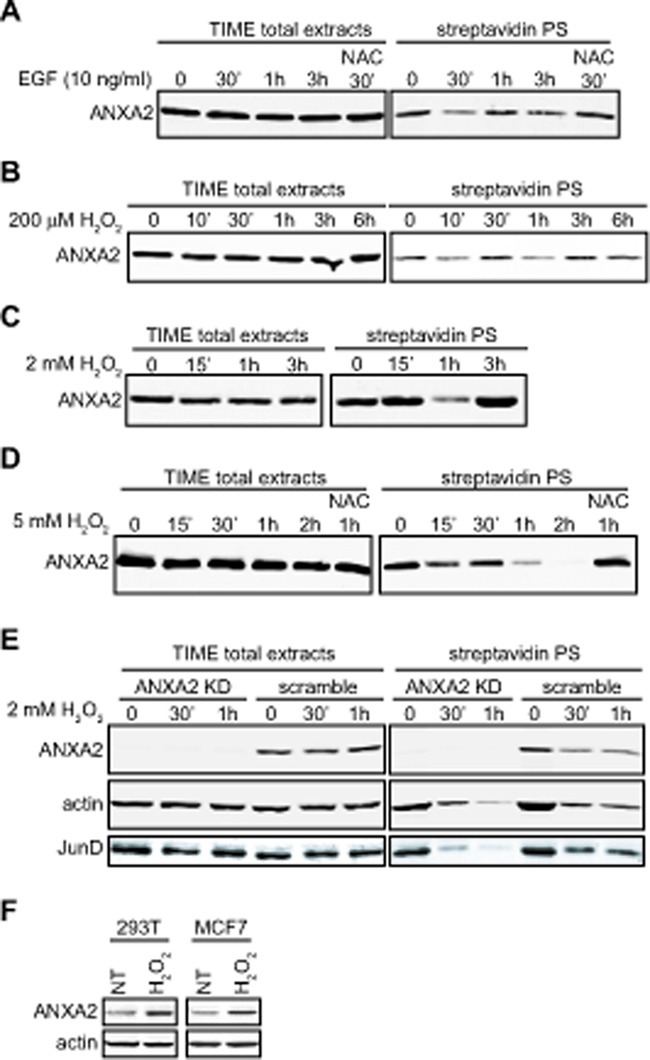
Cellular ANXA2 is responsive to reactive oxygen species (A) TIME cells were treated with 10 ng/ml EGF in the absence or presence of NAC for the times indicated; (B) TIME cells were treated with 200 μM H_2_O_2_ for the times indicated; (C) TIME cells were treated with 2 mM H_2_O_2_ for the times indicated; (D) TIME cells were treated with 5 mM H_2_O_2_ in the absence or presence of 10 mM NAC for the times indicated; (E) TIME ANXA2 shRNA2 (KD) or TIME ANXA2 scramble cells were treated with 2 mM H_2_O_2_ for the times indicated. (A-E) Lysates were labeled with 10 μM BIAM and 200 μg of each labeled lysate was incubated with streptavidin beads. Total cell extracts and streptavidin purified samples (streptavidin PS) were subjected to SDS-PAGE followed by western blotting with the antibodies indicated. (F) 293T and MCF7 cells were treated with 100 μM H_2_O_2_ for two weeks. 20 μg of each cell lysate was subjected to SDS-PAGE followed by western blotting with the antibodies indicated.

In order to test if ANXA2 plays a role in cellular redox regulation we investigated if this protein is sensitive to H_2_O_2_-induced oxidative stress. We first examined ANXA2 sensitivity to a physiological stimulus that produced small localized increases in intracellular H_2_O_2_. The interaction of epidermal growth factor (EGF) with its receptor stimulates the activity of the NADPH oxidase, Nox resulting in a rapid and transient increase in intracellular H_2_O_2_ levels to nanomolar concentrations [18]. In order to determine if ANXA2 was oxidized by EGF-generated H_2_O_2_ we took advantage of the selective reactivity of Biotin-conjugated iodoacetamide (BIAM) with the reactive thiols of proteins. BIAM has been commonly used to quantify redox sensitive cysteine oxidation by ROS since it selectively reacts with redox sensitive cysteine(s) (Cys-S^−^) at physiological pH, but not with Cys-SH or oxidized cysteine residues (Cys-SOH or Cys-S-S-Cys) [19, 20]. A decrease in BIAM labeling of proteins, as monitored by streptavidin blot analysis, indicates oxidation of the redox sensitive cysteine(s) by ROS. Accordingly, TIME cells were incubated with EGF and cellular extracts were either analyzed by SDS-PAGE followed by western blotting for ANXA2 or incubated with BIAM and the labeled/ biotinylated proteins purified by incubation with streptavidin beads, followed by SDS-PAGE and western blotting for ANXA2. Our results showed that ANXA2 was highly oxidized by 30 minutes after EGF stimulation. However, by 1 hour after treatment, we observed an up-regulation in the levels of reduced ANXA2. Pre-incubation of cells with the antioxidant agent N-acetyl cysteine (NAC) prevented the EGF-dependent oxidation of ANXA2 (Figure 1A), confirming that the EGF-dependent loss in the labeling of ANXA2 by BIAM was due to oxidation of ANXA2.

**Figure 2:**
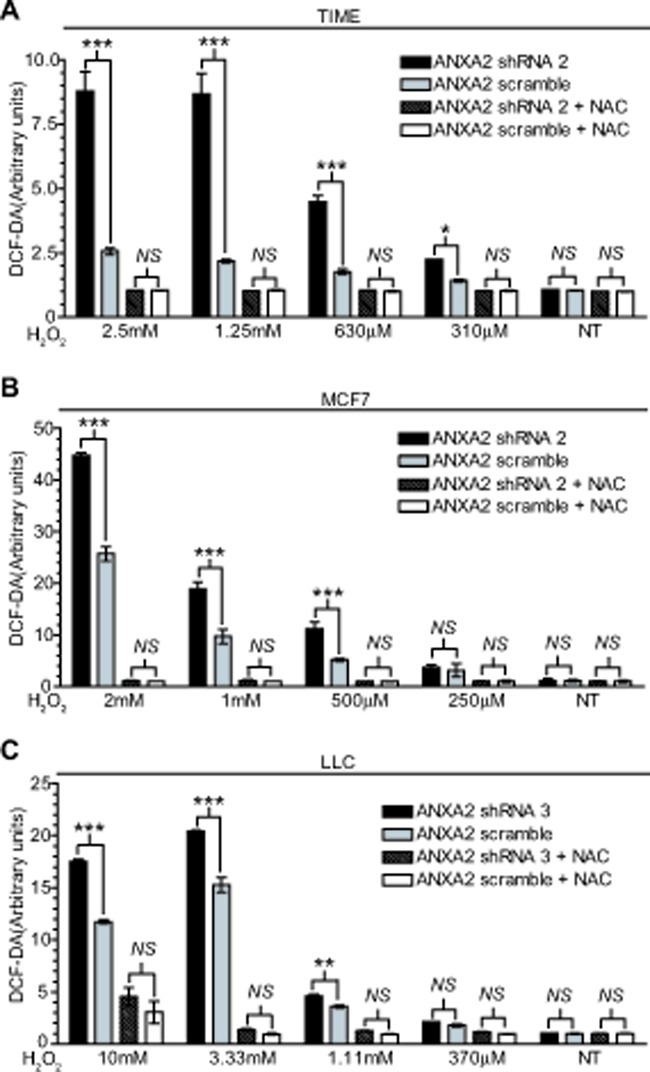
ANXA2 depleted cells accumulate higher levels of ROS upon oxidative stress (A) TIME ANXA2 shRNA 2 or TIME ANXA2 scramble cells, (B) MCF7 ANXA2 shRNA 2 or MCF7 ANXA2 scramble cells and (C) LLC ANXA2 shRNA 3 or LLC ANXA2 scramble cells were incubated with 50 μM DCF-DA reagent. Cells were treated with different concentrations of H_2_O_2_ in the absence or presence of 10 mM NAC. Fluorescence was measured using a fluorometer plate reader. Data are represented as ± StDev (N=4).

Next, we induced a slight increase in intracellular H_2_O_2_ levels by adding 200 μM H_2_O_2_ to the medium of TIME cells (see Figure 2A, grey bar), and observed that ANXA2 was transiently and reversibly oxidized by H_2_O_2_ (Figure 1B). Three hours after treatment with H_2_O_2_, the levels of reduced ANXA2 were similar to untreated cells. Importantly, the total levels of ANXA2 were unchanged during the time course of the experiment. This result suggested that ANXA2 was oxidized by H_2_O_2_ and this oxidation occurred concomitantly with small changes of intracellular H_2_O_2_. We also examined the redox status of ANXA2 during more extreme oxidative stress conditions. We observed ANXA2 oxidation one hour after adding 2 mM H_2_O_2_ to the medium of TIME cells. However, by three hours, the levels of reduced ANXA2 were similar to untreated cells (Figure 1C). These results show that high levels of H_2_O_2_ can also result in the reversible oxidation of ANXA2.

We investigated the redox status of ANXA2 under oxidative conditions that result in cell death. For these experiments, 5 mM H_2_O_2_ was added to the medium of TIME cells. The observed H_2_O_2_-dependent loss of BIAM labeling demonstrated that ANXA2 was oxidized by lethal concentrations of H_2_O_2_, however under these conditions, ANXA2 was irreversibly oxidized (Figure 1D). Pre-incubation of cells with the antioxidant NAC prevented the oxidation of ANXA2 (Figure 1D), again confirming that oxidation of ANXA2 by H_2_O_2_ was responsible for the loss of BIAM reactivity.

Although our results suggested that ANXA2 was a target for oxidation by H_2_O_2_ it was unclear if ANXA2 was involved in redox signaling or in redox regulation. Therefore, we generated stable ANXA2 knockdown cell lines using a retroviral vector-based shRNA system as shown in Figures 1E and S1 and confirmed that other annexins were not down-regulated in the ANXA2 depleted cells by western blot analysis (Figure S1). Next we increased intracellular H_2_O_2_ by addition of 2 mM H_2_O_2_ to the medium of ANXA2 depleted TIME and control cells. The cell extracts were incubated with BIAM and the BIAM-labeled proteins were collected by incubation with streptavidin beads, followed by SDS-PAGE and western blot analysis. We examined the redox status of two well described redox-sensitive proteins, namely JunD, a major oxidative stress responsive transcription factor and actin which is also highly sensitive to changes in intracellular ROS levels. As shown in Figure 1E, oxidation of both JunD and actin was more rapid and pronounced in ANXA2 depleted TIME cells compared to control cells suggesting that the intracellular levels of ROS were increased in ANXA2 depleted cells during oxidative stress. This result suggested that ANXA2 might regulate ROS levels during oxidative stress i.e. play a role in cellular redox regulation.

Several reports have shown that cells up-regulate antioxidant proteins in response to oxidative stress in order to maintain redox equilibrium and avoid cellular damage/death [21]. We investigated ANXA2 expression in response to a long term treatment with sub-lethal doses of H_2_O_2_. For this experiment we used cell lines that express ANXA2 at low levels, 293T cells, or moderate levels, MCF7 cells. These results showed up-regulation of ANXA2 expression in both cell lines under oxidative stress conditions, further supporting a role for ANXA2 as a redox regulatory protein (Figure 1F).

### ANXA2 depleted cells accumulate higher levels of ROS upon oxidative stress

Since our results suggested that ANXA2 might regulate ROS levels during oxidative stress we measured intracellular ROS levels using the probe, 2',7' dichlorodihydrofluorescein diacetate (DCF-DA) in either untreated or H_2_O_2_ treated cells. We noted that depletion of ANXA2 did not seem to significantly affect the basal levels of ROS in normal (TIME) or cancer cells (MCF7 and LLC) (Figure 2A-C). Strikingly, we observed that ROS levels were significantly higher in ANXA2 depleted cells after exposure to H_2_O_2_. Pre-incubation of cells with NAC blocked H_2_O_2_-induced increase in intracellular ROS, as expected. The levels of other redox regulatory proteins, including catalase, glutathione peroxidase, SOD-1, SOD-2, peroxiredoxins (I-IV) and thioredoxin were not altered in the ANXA2 depleted cells or in cells over-expressing ANXA2 as examined by western blotting (Figure S2). We also did not detect increases in the levels of glutathione (data not shown). Together these results demonstrate that ANXA2 plays a role in the regulation of ROS levels during oxidative stress.

### ANXA2 protects cells from oxidative stress induced death

**Figure 3:**
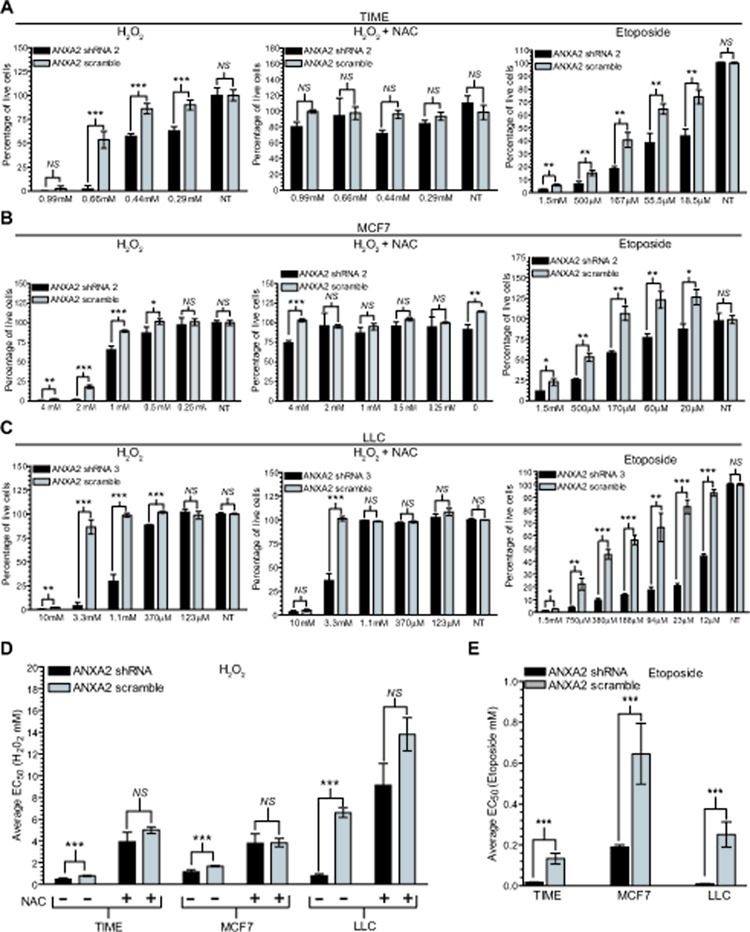
ANXA2 depleted cells are more sensitive to oxidative stress induced death (A) TIME ANXA2 shRNA2 or TIME ANXA2 scramble cells, (B) MCF7 ANXA2 shRNA2 or MCF7 ANXA2 scramble cells and (C) LLC ANXA2 shRNA 3 or LLC ANXA2 scramble cells were treated with different concentrations of H_2_O_2_ in the absence (left panel) or presence of 10 mM NAC (middle panel) for 24 hours or treated with etoposide (right panel) for 48 hours. Cell viability was measured using the CellTiter 96® AQueous Non-Radioactive Cell Proliferation Assay. Average 490 nm absorbance for non-treated (NT) cells was set as 100% viability. (D) Ec_50_ values for the cells treated with H_2_O_2_ in the absence or presence of NAC as indicated. (E) Ec_50_ values for the cells treated with etoposide. Data was analysed using the two tailed Student's t test and represented as ± StDev (N=4).

When oxidative stress exceeds the capacity of the cellular ROS scavenging system, cells succumb to the cytotoxic effects of the accumulated ROS and die [22, 23]. Since we observed increased levels of ROS in ANXA2 depleted versus control cells following oxidative stress, we hypothesized that ANXA2 might play a role protecting cells from ROS induced cell death. In order to investigate this possibility we analyzed the viability of ANXA2 depleted and control cells upon treatment with different concentrations of H_2_O_2_. We observed that cells depleted of ANXA2 were significantly more susceptible to H_2_O_2_-induced death compared to control cells (Figure 3A-C, left panels). The concentration of H_2_O_2_ that resulted in 50% loss in cell viability (EC_50_), decreased significantly in ANXA2 depleted cells compared to control cells (Figure 3D and table S1). Incubation of cells with NAC prior to treatment with H_2_O_2_ reversed this effect, as expected (Figure 3A-C, middle panels).

Many chemotherapeutic agents kill cancer cells through mechanisms that involve production of ROS. We observed that depletion of ANXA2 resulted in a significant increase in susceptibility to cell death induced by the ROS producing chemotherapeutic agent, etoposide (Figure 3A-C, right panels) [24]. The EC_50_ values were significantly lower in ANXA2 depleted cells compared to control cells (Figure 3E and table S1). We also observed a significant decrease in the EC_50_ values for ANXA2 depleted TIME and LLC cells treated with the chemotherapeutic doxorubicin and ANXA2 depleted MCF7 cells treated with tamoxifen (Figure S3 and Table S1).

### ANXA2 depleted cells show enhanced activation of oxidative stress responsive signaling proteins

After observing that ANXA2 depletion resulted in increased levels of ROS and loss of cell viability during oxidative stress, we investigated which oxidative stress responsive pathways were sensitive to the increased ROS levels observed in ANXA2 depleted cells. Cells typically use two main signaling pathways to respond to oxidative stress; the MAPK pathway that includes activation of the pro-apoptotic stress kinases c-Jun N-terminal kinases (JNKs) and p38 and the PI3K pathway, where activation of Akt by oxidative stress has been shown to be associated with apoptosis [25, 26].

**Figure 4:**
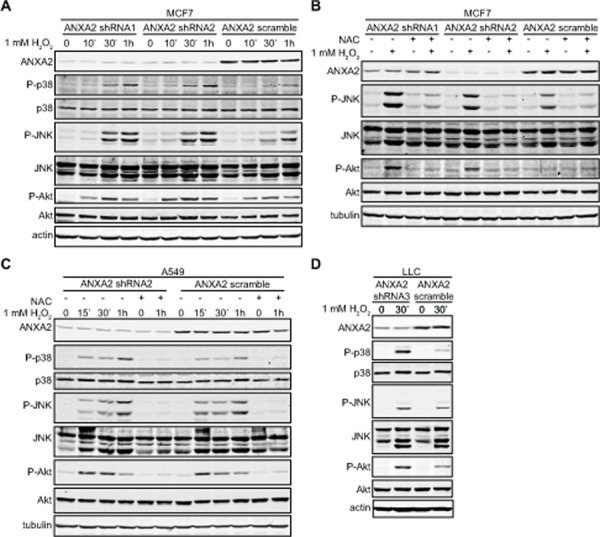
ANXA2 depleted cells show enhanced activation of oxidative stress responsive signaling proteins (A, B) MCF7 ANXA2 shRNA1, MCF7 ANXA2 shRNA2 or MCF7 ANXA2 scramble cells were treated with (A) 1 mM H_2_O_2_ for the times indicated or (B) 1 mM H_2_O_2_ in the absence or presence of 10 mM NAC for 30 min; (C) A549 ANXA2 shRNA2 or A549 ANXA2 scramble cells were treated with 1 mM H_2_O_2_ in the absence or presence of 10 mM NAC for the times indicated; (D) LLC ANXA2 shRNA3 or LLC ANXA2 scramble cells were treated with 1 mM H_2_O_2_ for 30 minutes. (A-D) 25 μg of each cell lysate was subjected to SDS-PAGE followed by western blotting with the antibodies indicated.

We treated ANXA2 depleted and control cells with H_2_O_2_ and subjected the cell extracts to SDS-PAGE followed by western blot analysis. The levels of phospho-p38 (P-p38), phospho-JNK (P-JNK) and phospho-Akt (P-Akt) prior to oxidative insult were similar in ANXA2 depleted and control cells, indicating that these pathways were not being activated simply by depletion of ANXA2. Interestingly, upon oxidative stress ANXA2 depleted cells showed significantly higher levels of P-p38, P-Akt and slightly elevated P-JNK compared to control cells (Figure 4), consistent with increased intracellular ROS levels and susceptibility of these cells to oxidative stress induced death. As predicted, pre-incubation of cells with NAC prevented the phosphorylation of these redox-responsive kinases, confirming that their activation was due to oxidative stress (Figure 4B, C).

### H_2_O_2_ oxidized ANXA2 is reversibly reduced by the Trx system

**Figure 5:**
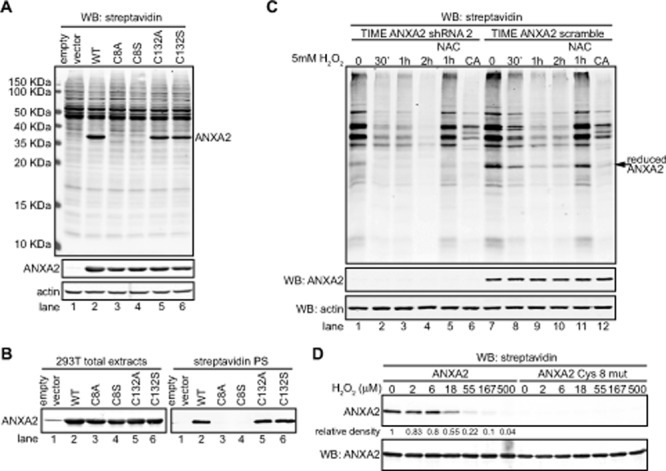
Cys^8^ residue of cellular ANXA2 is a redox sensitive cysteine (A) 293T cells were transiently transfected with pcDNA3 empty vector (lane 1), pcDNA3-ANXA2 WT (lane 2), or a series of ANXA2 cysteine mutant plasmids: pcDNA3-ANXA2-Cys-8-Ala (lane 3), pcDNA3-ANXA2-Cys-8-Ser (lane 4), pcDNA3-ANXA2-Cys-132-Ala (lane 5) or pcDNA3-ANXA2-Cys-132-Ser (lane 6) for 48 hours. 20 μg of each cell extract was labeled with 20 μM BIAM and subjected to SDS-PAGE followed by western blotting with a streptavidin probe and the antibodies indicated. (B) 200 μg of each cell lysate was incubated with 20 μM BIAM, labeled proteins were purified with streptavidin beads. Cell extracts and streptavidin purified samples (streptavidin PS) were subjected to SDS-PAGE followed by western blotting for ANXA2. (C) TIME ANXA2 shRNA2 or TIME ANXA2 scramble cells were treated with 5 mM H_2_O_2_ in the absence or presence of 10 mM NAC for the times indicated. CA stands for competition assay; these samples were treated with 5 mM DTT prior to incubation with BIAM. Cell extracts were labeled with 10 μM BIAM, subjected to SDS-PAGE followed by western blotting with the antibodies indicated. (D) 0.65 μM of human recombinant ANXA2 or ANXA2^C8S^ protein was incubated with the indicated concentrations of H_2_O_2_ for 30 minutes, after what 10 μg/ml of catalase was added. Samples were labeled with 20 μM BIAM and subjected to SDS-PAGE followed by western blotting with a streptavidin probe and ANXA2 antibody. Protein band quantification was done using the Licor Odyssey software.

Collectively, our data indicated that ANXA2 acted as a cellular redox regulatory protein; however the mechanism by which ANXA2 interacted with ROS was unclear. Therefore, we investigated which cysteine residue(s) of cellular ANXA2 were oxidized by ROS. Previous work from our laboratory has identified two cysteine residues of ANXA2, Cys^8^ and Cys^132^, as potential reactive residues based on their modification by glutathione *in vitro.* In order to further investigate which of these residues are redox sensitive cysteine(s) *in vivo*, we over-expressed ANXA2 wild-type (WT), and several cysteine mutants in 293T cells. These cells were chosen because they have low levels of endogenous ANXA2. The levels of over-expression of ANXA2 in the 293T cells were comparable to the endogenous levels of this protein in A549 breast cancer cells (Figure S4A) and lower than in the TIME cells (data not shown), indicating that ANXA2 over-expression in the 293T cells resulted in intracellular levels of ANXA2 were similar to the levels of ANXA2 observed in other cell lines. We incubated cell lysates from the 293T cells with BIAM, followed by SDS-PAGE and western blotting with a streptavidin probe, in order to identify BIAM labeled proteins. We observed that both ANXA2^C8S^ and ANXA2^C8A^ proteins were expressed in the 293T cells at comparable levels to ANXA2 WT, ANXA2^C132S^ and ANXA2^C132A^. However, the ANXA2 Cys^8^ mutant proteins were not labeled by BIAM. In contrast, the labeling of ANXA2^C132S^ and ANXA2^C132A^ was similar to that of ANXA2 WT (Figure 5A). This result shows that only Cys^8^ and not Cys^132^ of ANXA2 is a redox sensitive cysteine *in vivo*. Furthermore, we also observed that ANXA2 was one of the main BIAM labeled bands in the whole cell extracts, indicating that the Cys^8^ residue of ANXA2 is highly reactive/ sensitive to oxidation. Next, we repeated this experiment, but collected the BIAM labeled proteins with streptavidin beads and performed a western blotting for ANXA2. As shown in Figure 5B we were unable to detect the ANXA2 Cys^8^ mutants. This established that Cys^8^ of ANXA2 is the redox sensitive cysteine.

In order to determine the relative abundance of ANXA2 compared with other redox sensitive proteins, we labeled the cell extracts of ANXA2 depleted and control TIME cells with BIAM, followed by SDS-PAGE and western blotting with a streptavidin probe (Figure 5C). We did not detect a BIAM labeled band around 36 KDa (ANXA2 molecular weight) in the ANXA2 depleted TIME cells, and ANXA2 was one of the major proteins that reacted with BIAM in the TIME control cells, indicating the abundance of ANXA2 and the high reactivity of its Cys^8^ redox sensitive cysteine in these cells. Furthermore, we observed that oxidative stress resulted in a more dramatic and rapid oxidation of cellular proteins in the ANXA2 depleted TIME cells compared to control cells (Figure 5C), further suggesting that ANXA2 plays a role in redox regulation. Pre-incubation of cells with NAC prevented the oxidation of ANXA2 (Figure 5C, lane 11). As an additional control we observed that addition of 5 mM DTT to the cell extracts prior to incubation with BIAM abolished BIAM labeling of ANXA2. Since DTT competes with ANXA2 for the BIAM reagent, this result establishes that ANXA2 biotinylation is due to BIAM labeling of its redox sensitive cysteine and that ANXA2 does not exist as a biotinylated protein in the cells (Figure 5C, lane 12).

The reagent 3-(N-Maleimidylpropionyl)biocytin (NEM-biotin), although distinct in chemical reactivity from BIAM is also commonly used to identify redox sensitive cysteines in proteins [27]. We observed in experiments in which cell extracts were labeled with NEM-biotin that ANXA2 with C8S and C8A substitutions completely failed to react with this probe, while ANXA2 with C132S and C132A substitutions reacted with this probe similarly to ANXA2 WT protein (Figure S4B). This result further supported that Cys^8^ residue is the only redox sensitive cysteine of ANXA2.

H_2_O_2_ is a key ROS that also functions as a second messenger molecule in cells. To investigate whether the redox sensitive cysteine of ANXA2, Cys^8^, is a direct target for H_2_O_2_ oxidation, we incubated human recombinant ANXA2 with increasing amounts of H_2_O_2_, quenched the reaction with catalase and labeled the protein with BIAM followed by SDS-PAGE and western blotting with a streptavidin probe. These results showed that ANXA2 reacted with BIAM and that the BIAM labeling of ANXA2 decreased with increasing concentrations of H_2_O_2_ establishing that ANXA2 was oxidized by H_2_O_2_ in a dose dependent manner (Figure 5D). However, the ANXA2^C8S^ mutant protein did not react with the BIAM reagent as expected, confirming again that Cys^8^ residue is the redox sensitive cysteine of ANXA2 (Figure 5D).

**Figure 6:**
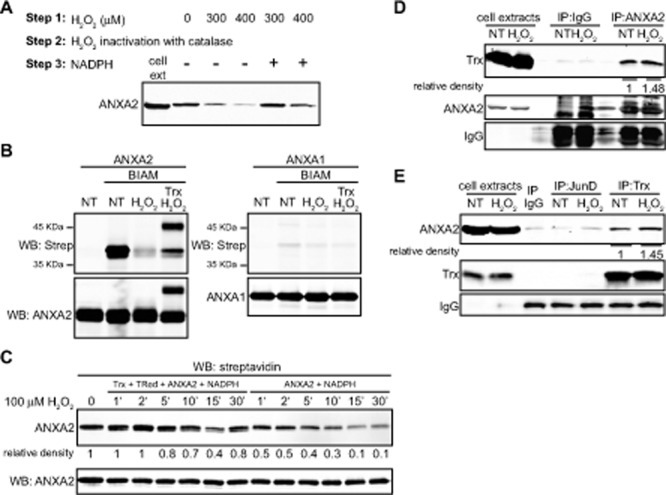
ANXA2 is a substrate of the Trx system (A) TIME cell extracts were treated with the indicated amounts of H_2_O_2_, incubated with catalase after what mock or 500 μM of NADPH was added, as indicated. Lysates were incubated with 10 μM BIAM and labeled proteins were purified with streptavidin beads. Cell extracts of non treated cells (lane 1) and streptavidin purified samples were subjected to SDS-PAGE followed by western blotting for ANXA2. (B) 2.5 μM ANXA2 or ANXA1 were incubated in the absence or presence of 100 μM H_2_O_2_ with or without 20 μM of Trx as indicated. Proteins were incubated in the absence or presence of 20 μM BIAM, subjected to SDS-PAGE under non-reducing conditions, followed by western blot analysis with the antibodies indicated. (C) 2 μM of human recombinant ANXA2 were incubated with 0.15 μM Trx reductase, 0.5 μM Trx, 500 μM NADPH (left panel) or with NADPH alone (right panel). 100 μM of H_2_O_2_ was added and samples were collected at the times indicated, after what 10 μg/ml of catalase was added. Samples were labeled with 20 μM BIAM and subjected to SDS-PAGE followed by western blotting with a streptavidin probe. (D) A549 cells were either non-treated (NT) or treated with 1 mM H_2_O_2_ for 1 hour. Cells were lysed and immunoprecipitated with IgG, anti-JunD or anti-Trx antibodies as indicated; (E) A549 cells were either non-treated (NT) or treated with 1 mM H_2_O_2_ for 1 hour. Cells were lysed and immunoprecipitated with either IgG or anti-ANXA2 antibodies as indicated (E) A549 cells were either non-treated (NT) or treated with 1 mM H_2_O_2_ for 1 hour. Cells were lysed and immunoprecipitated with IgG, anti-JunD or anti-Trx antibodies as indicated. (D-E) Immunoprecipitates and 20 μg of each cell extract were subjected to SDS-PAGE followed by western blotting with the antibodies indicated. Protein band quantification was done using the Licor Odyssey software.

The NADPH-dependent thioredoxin and glutathione redox systems are the best characterized antioxidant systems that are utilized by the cells to reduce/restore oxidized proteins. We observed that incubation of a cellular lysate with H_2_O_2_ caused the oxidation of ANXA2 and that the subsequent addition of NADPH to this cellular lysate reversed ANXA2 oxidation (Figure 6A). This result shows that ANXA2 can be reversibly oxidized by H_2_O_2_ and reduced by an NADPH-dependent antioxidant system in the cells. We next investigated if the Trx antioxidant system was involved in the restoration of oxidized ANXA2 to its reduced form. Human recombinant ANXA2 was incubated in the presence or absence of H_2_O_2_ and the sample was labeled with BIAM. As expected, incubation of ANXA2 with H_2_O_2_ resulted in the oxidation of ANXA2. Interestingly, if the oxidized ANXA2 was then incubated with thioredoxin, both ANXA2 and a 48 KDa complex of ANXA2 and thioredoxin were observed by SDS-PAGE and western blotting under non-reducing conditions. Furthermore, the formation of the ANXA2-thioredoxin complex resulted in the reduction of H_2_O_2_-oxidized ANXA2 (Figure 6B). As a control we repeated this experiment with ANXA1, an annexin closely related to ANXA2. ANXA1 does not possess a redox sensitive cysteine and the BIAM reagent did not label this protein. Since ANXA1 contains cysteine residues this result further supports the specific labeling of reactive thiols by BIAM (Figure 6B). We also observed that when human recombinant ANXA2 was incubated with 100 μM H_2_O_2_ and NADPH (control), the ANXA2 was rapidly oxidized by 50% within one minute and by 90% after 30 minutes of incubation with H_2_O_2_. However, the inclusion of Trx reductase, Trx and NADPH (the Trx antioxidant system) resulted in the reduction of H2O2-oxidized ANXA2 (Figure 6C). ANXA2 reduced form was similar to non treated protein until 5 minutes after addition of H_2_O_2_, at 10 minutes post-incubation with H_2_O_2_ we observed a mild oxidation of ANXA2, around 30%, which increased by 15 minutes post-incubation to 60%, but ANXA2 reduced levels were almost completely restored by 30 minutes after incubation with 100 μM H_2_O_2_ in the presence of the Trx redox system. Together these results indicate that H_2_O_2_-oxidized ANXA2 is reversibly reduced by the Trx system.

In order to determine if ANXA2 interacts with Trx *in vivo* we performed co-immunoprecipitation studies. Cells were incubated in the absence or presence of H_2_O_2_ and the cell lysates were immunoprecipitated with antibodies against ANXA2, Trx or JunD (control). These results confirmed that Trx and ANXA2 formed a complex whose levels increased 1.5 fold after stimulation with H_2_O_2_ (Figure 6D, E).

### ANXA2-null mice show increased protein oxidation in the liver and lungs

**Figure 7:**
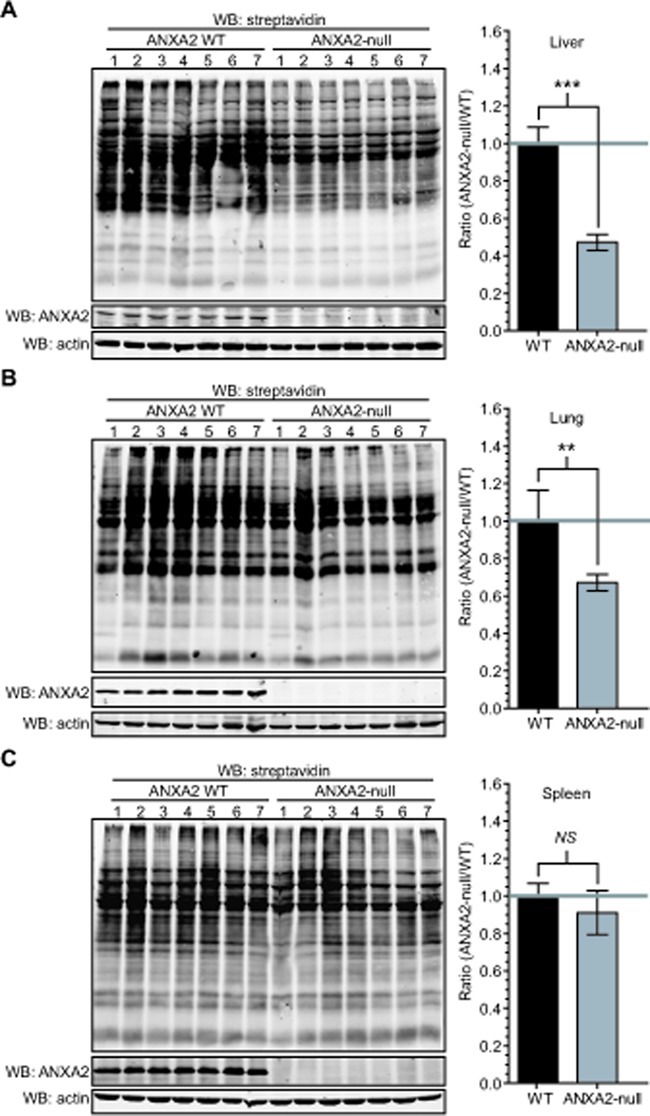
ANXA2-null mice show increased protein oxidation in the liver and lungs ANXA2 WT or ANXA2-null mice age matched were sacrificed, (A) livers, (B) lungs and (C) spleens were removed, homogenized and labeled with 20 μM BIAM. Samples were subjected to SDS-PAGE followed by western blotting with a streptavidin probe (left panels). The right panels show the quantification of the results on the left panels using the Licor Odyssey software. Data was analyzed using the two tailed Student's t test and represented as ± StDev (N=7).

We used ANXA2-null mice to study the role of ANXA2 in cellular redox regulation *in vivo* [28]. We reasoned that if ANXA2 played a role in cellular redox regulation its loss would result in enhanced oxidative stress which could be visualized by increased tissue protein oxidation. In order to investigate this possibility, we removed and homogenized organs from age-matched WT and ANXA2-null mice, labeled the cell extracts with BIAM, followed by SDS-PAGE and western blotting with a streptavidin probe. We observed a 50% decrease in the BIAM labeling of liver proteins in ANXA2-null mice compared to WT mice (Figure 7A) and a 30% decrease in BIAM labeling of lung proteins in ANXA2-null mice (Figure 7B). These data established that the liver and lung tissues of ANXA2-null mice were significantly more oxidized compared to WT mice. Interestingly, the oxidation status of spleen proteins was similar in the ANXA2-null and WT mice (Figure 7C).

### ANXA2 redox regulatory function plays a role in tumorigenesis

**Figure 8:**
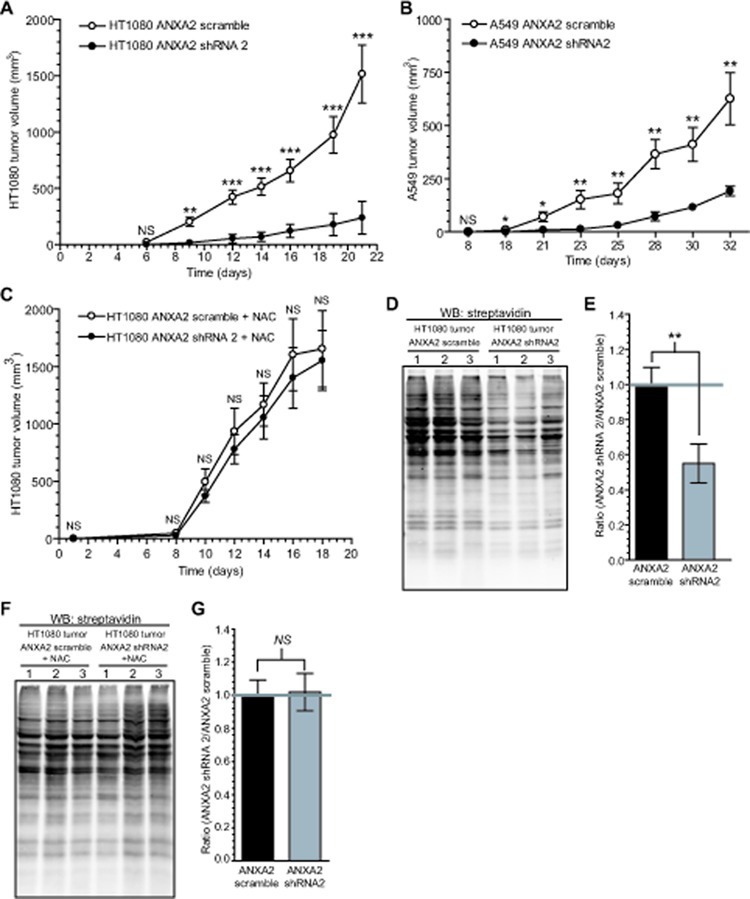
ANXA2 redox regulatory function plays a role in tumorigenesis (A) 2 X 10^6^ HT1080 ANXA2 shRNA2 or HT1080 ANXA2 scramble cells (N=10 per group) (B) 2 X 10^6^ A549 ANXA2 shRNA2 or A549 ANXA2 scramble cells (N=8 per group) were injected subcutaneously into the right flank of NOD-SCID mice. (C) 2 X 10^6^ HT1080 ANXA2 shRNA2 or HT1080 ANXA2 scramble cells were injected subcutaneously into the right flank of NOD-SCID mice treated with intraperitoneal injections of 150 mg/kg of NAC every two days (N=8 per group). (A-C) Tumor volume was measured for the time indicated using a caliper. Data was analyzed using the 2-way Analysis of Variance (ANOVA) test and represented as ± SEM. (D-G) Three HT1080 ANXA2 shRNA2 and three HT1080 ANXA2 scramble tumors from mice untreated (D) or treated (F) with NAC were homogenized, labeled with 20 μM BIAM, subjected to SDS-PAGE and analyzed by western blotting with a streptavidin probe. (E) and (G) Quantification of the results obtained in (D) and (F) using the Licor Odyssey software, respectively.

Tumors are typically under higher oxidative stress compared to neighboring normal cells. We reasoned that if ANXA2 plays a role in cellular redox regulation its depletion will render cancer cells more susceptible to ROS induced damage/death during tumorigenesis. In order to investigate this hypothesis, we established ANXA2 depleted HT1080 and A549 cells and respective control cells and analyzed the early passaged cells for protein oxidation. As assessed by BIAM labeling, we observed no difference in the oxidation of cellular proteins in ANXA2 depleted versus control cell lines while cultured in optimal tissue culture conditions (Figure S5A, F). This was expected as these cells were not under oxidative stress. The ANXA2 depleted and control cells were injected subcutaneously into the right flank of NOD-SCID mice and tumor growth was monitored by caliper measurement. We observed a significant impairment of tumor growth by ANXA2 depleted HT1080 and A549 cancer cells compared to control cells (Figure 8A, B). At the experimental end point, the volume of tumors grown from ANXA2 depleted HT1080 cells was approximately 85% smaller compared to tumors grown from control HT1080 cells. Furthermore, the volume of tumors grown from ANXA2 depleted A549 cancer cells was 70% smaller compared to A549 control tumors. The growth impairment of ANXA2 depleted A549 and HT1080 tumors was not due to decreased cell proliferation since we observed the same growth rate for ANXA2 depleted and control cell lines in culture (Figure S5E, H). In order to investigate if the growth impairment observed for the ANXA2 depleted tumors was due to ROS overload, we repeated the tumor growth experiments using ANXA2 depleted HT1080 and control cells and administered the antioxidant agent, NAC intraperitoneally every two days. These data showed that the growth rate of the ANXA2 depleted HT1080 tumors in mice receiving NAC injections was only slightly lower than the HT1080 control tumors (Figure 8C). At the end point of the experiment the ANXA2 depleted HT1080 tumor volume was only 8% smaller than the HT1080 control tumors. To investigate the redox status of the cellular proteins in the ANXA2 depleted HT1080 or control tumors in the absence or presence of NAC injections, we removed HT1080 tumors from untreated or NAC treated mice and labeled the cellular extracts with BIAM. The tumors grown from ANXA2 depleted HT1080 cells showed a 45% decrease in protein labeling (increased oxidation) compared to tumors grown from control HT1080 cells (Figure 8D, E). However, in mice receiving NAC injections, the labeling of tumor extracts by BIAM suggested similar levels of protein oxidation between tumors grown from ANXA2 depleted or control HT1080 cells (Figure 8F, G). These results indicate that NAC prevented the intracellular protein oxidation observed in tumors grown from ANXA2 depleted HT1080 cells. We examined the redox status of protein extracts obtained from ANXA2 depleted HT1080 and control cells that were grown in culture for the time course of the animal experiments. Interestingly, we observed no difference in the levels of BIAM labeling between ANXA2 depleted HT1080 and control cells that were grown in tissue culture conditions, under no oxidative stress, for three weeks (Figure S5B). This suggested that the oxidative stress that is known to occur during tumor growth, had a more severe effect on the ANXA2 depleted HT1080 cells than the control HT1080 cells. Collectively, these results support a role for ANXA2 in cancer cell redox regulation during tumorigenesis.

### ANXA2 redox status in human tumors

**Figure 9:**
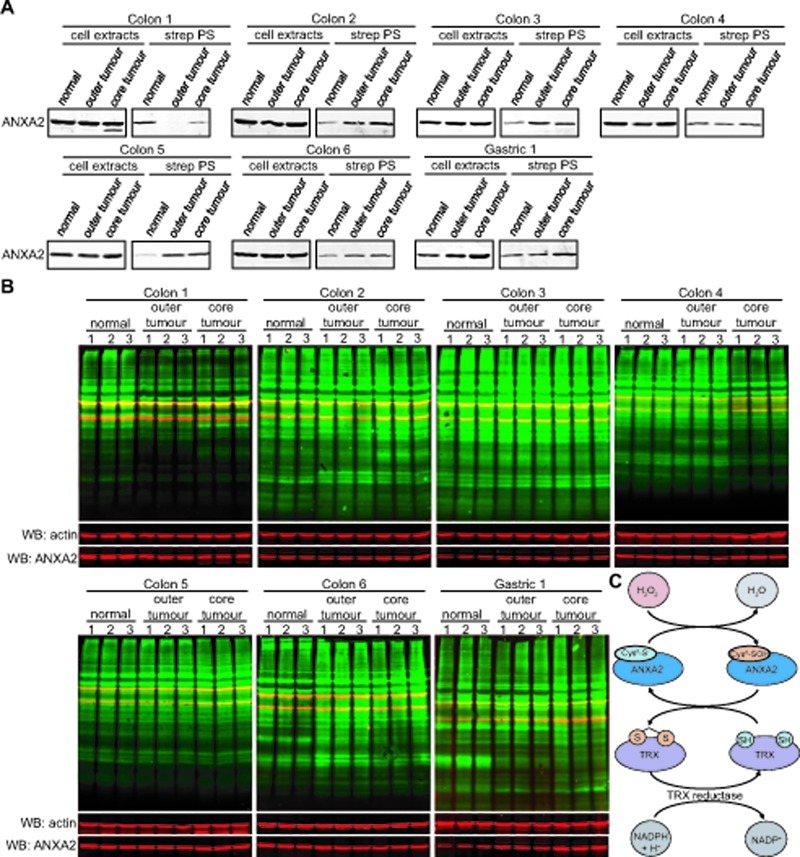
ANXA2 redox status in human normal and tumor tissues (A,B) Six different sets of human colon tissue extracts and one set of gastric tissue extracts, including normal tissue, outer tumor and core tumor were labeled with 20 μM BIAM; (A) Labeled extracts were purified by incubation with streptavidin beads. Cell extracts and streptavidin purified samples (strep PS) were subjected to SDS-PAGE followed by western blot analysis with the antibodies indicated; (B) 20 μg of each labeled cell extract were subjected to SDS-PAGE, followed by western blotting with a streptavidin probe and the antibodies indicated. (C) H_2_O_2_ oxidizes Cys^8^ of ANXA2 resulting in the conversion of H_2_O_2_ to H_2_O. Oxidized ANXA2 is then reduced by the Trx redox system and can participate in further redox cycles. Thus a single molecule of ANXA2 can degrade several molecules of H_2_O_2_.

We also investigated the redox status of ANXA2 in colon and gastric tumors compared to adjacent normal tissue. We examined six different human colon sample sets and one gastric set, including: normal tissue, outer tumor and core tumor. Cell extracts were labeled with BIAM and purified with streptavidin beads as described previously. We observed that the total levels of ANXA2 were unchanged in all colon samples and moderately elevated in the gastric tumor. However, the levels of the reduced form of ANXA2 were up-regulated in the gastric tumor and in four out of the six colon tumors compared to normal tissues (Figure 9A). Furthermore, in the two sets of colon samples where this did not occur, we observed significant oxidation of total proteins in the tumors (Figure 9 B- colon 1, 4). Colon 1 showed significantly elevated oxidation of total proteins in the outer and core tumors consistent with the high level of ANXA2 oxidation in these samples, whereas in colon 4 where the levels of reduced ANXA2 were similar in normal and tumor samples we only observed a significant oxidation of total proteins in the core/hypoxic tumor, which is under higher oxidative stress due to lack of blood supply.

## DISCUSSION

In the current study we identify ANXA2 as a novel cellular redox regulatory protein that plays a significant role in cells undergoing oxidative stress and in particular during tumorigenesis.

With this work we show for the first time that Cys^8^ residue of ANXA2 is a redox sensitive cysteine that is reversibly oxidized by H_2_O_2_ and reduced by the thioredoxin system. Our results allow us to propose the following mechanistic model: H_2_O_2_ reacts with the Cys^8^ of ANXA2 resulting in the oxidation of this residue and the conversion of H_2_O_2_ to H_2_O. Oxidized ANXA2 is then reduced by the Trx redox system and can participate in further redox cycles (Figure 9C). Thus a single molecule of ANXA2 can degrade several molecules of H_2_O_2_.

Considering the abundance and wide distribution of ANXA2 in cells and the fact that ANXA2 contains a highly reactive cysteine residue that is a direct target of H_2_O_2_, we hypothesized that ANXA2 might play an important role in the cellular response to oxidative stress, particularly H_2_O_2_-mediated stress. As mentioned before, H_2_O_2_ is a main second messenger in cell signaling transduction and is induced by a number of signaling pathways that regulate cell proliferation, survival and apoptosis. A tight control in the levels of cellular H_2_O_2_ is then very important for cell signaling regulation and in order to avoid protein, lipid and DNA oxidative damage. Here we show that ANXA2 depleted cells exhibit significantly enhanced levels of ROS and increased oxidation of redox sensitive proteins upon H_2_O_2_ treatment compared to control cells. Although we did not observe differences in the basal levels of ROS in untreated ANXA2 depleted versus control cells using the DCF-DA reagent, it is possible that the DCF-DA reagent is not sensitive enough to detect small differences in the basal concentrations of cellular H_2_O_2_ using a fluorometer plate reader. Alternatively, it is possible that ANXA2 only plays a significant redox regulatory role under oxidative stress conditions, while under physiological conditions other antioxidant proteins are able to compensate for the loss of ANXA2.

The enhanced ROS levels observed in the ANXA2 depleted cells upon oxidative stress resulted in increased sensitivity of these cells to ROS induced cell death compared to control cells. Chemotherapeutic agents that induce oxidative stress are thought to selectively kill cancer cells because the elevation of ROS caused by these agents is less toxic to normal cells that have lower levels of endogenous ROS. However, we surprisingly found that our non cancer cell model was more sensitive to oxidative stress compared to cancer cells. Nevertheless, these are different cell types and for this reason it is not appropriate to directly compare their sensitivity to ROS induced death. Furthermore, redox adaptation by cancer cells can in some cases make these cells more resistant to ROS induced death. For instance it has been shown that the MCF10 normal breast cells are more sensitive to H_2_O_2_ induced cell death compared to MCF7 breast cancer cells, because MCF7 cells up-regulate the peroxidases, peroxiredoxin I and II in order to counteract the elevation of ROS and prevent cellular damage/death [29]. Interestingly, we observed that ANXA2 protein levels increased by two-fold after treatment of 293T and MCF7 cells with 100 μM H_2_O_2_ for two weeks. This result suggests that cells might respond to elevations in ROS levels/ oxidative stress by inducing the expression of the redox regulatory protein, ANXA2.

The observed increased sensitivity of ANXA2 depleted cells to death induced by chemotherapeutics that up-regulate ROS led us to postulate that blocking the expression of ANXA2 in cancer cells might constitute a potential therapeutic strategy. Several reports have shown that up-regulation of ANXA2 in tumors correlates with chemoresistance, but they were unable to explain the mechanism(s) by which this occurs. Our results provide an explanation for this observation, since ANXA2 antioxidant function will protect the cancer cells from oxidative damage/death induced by the chemotherapeutics.

We also analyzed the activation of H_2_O_2_ responsive signaling pathways in the ANXA2 depleted versus control cells. The major intracellular sensor of oxidative stress induced by H_2_O_2_ is the apoptosis signal-regulated kinase-1 (ASK-1). ASK-1 directly activates MKK3/MKK6 and MKK4/MKK7 kinases which phosphorylate the pro-apoptotic kinases, p38 and JNK [30, 31, 32]. Therefore, the phosphorylation status of p38 and JNK provides a sensitive readout of the redox status of the cell. Elevated levels of H_2_O_2_ also lead to activation of the PI3K pathway kinase, Akt through inactivation of PTEN phosphatase [33]. Although Akt signaling is usually associated with cell survival, recently it has been shown that under oxidative stress conditions Akt further increases the intracellular levels of ROS through an increase in oxygen consumption and inhibition of FoxO transcription factors sensitizing cells to ROS-mediated apoptosis [26]. We observed enhanced activation of the pro-apoptotic kinases p38, JNK and Akt in ANXA2 depleted cancer cells treated with H_2_O_2_ compared to control cells. These results provide a molecular mechanism that explains the increased sensitivity of these cells to ROS induced death.

If ANXA2 functions as a redox regulatory protein then it is reasonable to expect that the ANXA2-null mice would exhibit a phenotype. However, the cellular redox system is complex with significant cross talk between various proteins and often several proteins compensate for each others function in a relatively efficient way under non pathological conditions. Considering the importance of cellular redox regulation it is not surprising that cells possess redox proteins with overlapping functions. For instance, homozygous catalase-null mice, which are completely deficient in catalase expression, the major cellular enzyme that decomposes H_2_O_2_, develop normally and show no gross abnormalities under physiological conditions. In fact, mice deficient in catalase were not more vulnerable to hyperoxia-induced lung injury; nor did their eye lenses showed any increased susceptibility to oxidative stress generated by a photochemical reaction,suggesting that the antioxidant function of catalase in these two models of oxidative injury is either negligible, which is unlikely, or is compensated by other cellular antioxidant protein(s) [34]. The only phenotype observed in these knockout mice was a retarded rate of breakdown of H_2_O_2_ by liver, lung and lens tissue slices compared to the wild-type mice.

Our animal studies showed that ANXA2-null mice have enhanced liver and lung protein oxidation compared to WT mice. Liver cells have a high metabolic rate since this organ is involved in protein, lipid and carbohydrate metabolism, synthesis of urea and the manufacture of bile, resulting in increased ROS production by the mitochondria. The lung is constantly exposed to oxidative stress due to gas exchange between the alveoli and the red blood cells. It is not surprising that depletion of the redox regulatory protein, ANXA2 in these organs will lead to increased oxidative stress. We did not observe significant differences in spleen protein oxidation between ANXA2-null and WT mice. This could be due to the fact that most splenocytes show a rapid turnover and are unlikely to accumulate as much ROS induced protein damage as the long-lived liver or lung cells.

During tumorigenesis, cancer cells typically exhibit increased ROS generation. In order to balance the proliferative advantages of ROS up-regulation versus its potential risks due to protein, lipid and DNA damage, cancer cells induce the over-expression and/or activation of antioxidant/redox regulatory proteins. Our *in vivo* tumor studies showed that tumor growth in animals injected with either ANXA2 depleted HT1080 or ANXA2 depleted A549 cancer cells was severely impaired compared to tumor growth in animals injected with control HT1080 and A549 cancer cells, respectively. In order to investigate if the growth impairment of ANXA2 depleted tumors was specifically due to ANXA2 redox regulatory function we treated mice possessing ANXA2 depleted HT1080 tumors, with the antioxidant NAC. We observed that in the presence of NAC tumor growth in animals injected with ANXA2-depleted cancer cells was identical to tumor growth in animals injected with control cancer cells. These results show that replacement of ANXA2 by another antioxidant reagent, such as NAC, reversed the growth impairment phenotype observed for the ANXA2 depleted tumors. It is then reasonable to propose that ANXA2 depletion from the cancer cells resulted in a loss of cellular redox regulatory capability which became critical as these cancer cells grew into tumors and were subjected to oxidative stress in the tumor site. The loss of ANXA2-dependent redox regulation during tumor growth was restored by the antioxidant activity of NAC. Consistent with this rationale was the observation that in the absence of NAC, the HT1080 ANXA2 depleted tumors showed significantly enhanced protein oxidation (sensor for cellular oxidative damage) compared to control tumors, while there was no enhanced protein oxidation in the ANXA2 depleted tumors grown in mice that received the antioxidant NAC. Taking these data together we therefore propose that ANXA2 plays a major role in tumorigenesis by functioning as a redox regulatory protein.

It was interesting to observe that NAC by itself stimulated tumor growth of the control cancer cells. This result is consistent with the reports that compared to normal cells, cancer cells are under significant oxidative stress and that agents that increase cellular ROS levels can selectively kill cancer cells [35, 36]. In this situation NAC can act by lowering the levels of H_2_O_2_ in the HT1080 tumors, thus increasing cell proliferation or decreasing cell death. This result also indicates that oxidative stress plays a major role and is a limiting step in tumor growth under our experimental conditions.

Although controversial, ANXA2 has also been implicated in other cellular processes, in particular plasmin activation, which could also contribute to cancer progression [37]. Several studies have shown that plasmin activation does not play a major role in the subcutaneous growth of primary tumors. It has been shown that sub-cutaneous inoculation of plasminogen negative tumour cells (LLC) in plasminogen-null mice produced tumors only 10-35% smaller compared to control mice [38], while we observed a 70-85% decrease in tumor volume in the A549 and HT1080 ANXA2 depleted tumors compared to control tumors. Another study showed that both WT and plasminogen-null mice carrying the Polyoma middle T antigen under the control of the mouse mammary tumor virus long terminal repeat uniformly developed multiple bilateral mammary tumors that were indistinguishable [39]. More importantly, plasmin generation by ANXA2 or other plasminogen receptors is not regulated by NAC, but is dependent on the presentation of a C-terminal lysine residue by the plasminogen receptor [40, 17, 37]. Consequently, if the growth deficit observed in the ANXA2 depleted tumors was due to decreased plasmin activation, the addition of NAC would not reverse the phenotype and restore the growth of ANXA2 depleted tumors as observed.

Finally, our *ex-vivo* experiments using human colon and gastric samples showed that five out of the seven sets of samples analyzed exhibited up-regulation of the reduced form of ANXA2 in the tumors compared to normal tissue. Whereas the two tumor samples where we did not observe up-regulation of reduced ANXA2 showed increased total protein oxidation. These results show that ANXA2 antioxidant function is important in maintaining redox equilibrium in the human tumors, since in general tumors show elevated levels of reduced ANXA2 and loss of reduced ANXA2 in tumors led to increased protein oxidation/oxidative stress.

In this study, we show that although ANXA2-null mice do not have an overt phenotype under non-pathological conditions, ANXA2 plays an important role during disease, namely supporting cancer progression by acting as a redox regulatory protein. Our observations that liver and lung tissue proteins from ANXA2-null mice are more oxidized compared to WT mice and that the proteins extracted from tumors produced by ANXA2-depleted cancer cells are more oxidized than proteins extracted from tumors produced by control cancer cells are also consistent with our proposed function for ANXA2 as an antioxidant.

Several reports have shown that up-regulation of ANXA2 levels is positively associated with cancer progression and chemoresistance, nevertheless the function of ANXA2 during these processes remained unknown. This is the first report that elucidates a molecular mechanism by which ANXA2 contributes to tumorigenesis and resistance to chemotherapy, by acting as a redox regulatory protein. Development of strategies that are aimed at preferentially killing cancer cells through mechanisms that cause additional ROS overload are currently being used. Agents that block ANXA2 expression could therefore be useful to preferentially kill malignant cells.

## MATERIALS AND METHODS

### Cell Culture, transfections and cell lines

MCF-7, A549, HT1080, LLC and 293T cell lines were obtained from ATCC and maintained in Dulbecco's modified Eagle's medium (Invitrogen) supplemented with 10% fetal bovine serum (FBS) and 100 U/ml of penicillin/streptomycin, in a humidified incubator in an atmosphere of 5% CO_2_ at 37^°^C. TIME endothelial cells were obtained from Dr McMahon [41] and maintained in EGM2 medium (Lonza) supplemented with 10% FBS and 100U/ml of penicillin/streptomycin, in a humidified incubator in an atmosphere of 5% CO_2_ at 37^°^C. ANXA2 depleted cell lines were obtained by transfection of Phoenix packaging cells with 4 μg of the pSUPER-retro plasmids described below using 12 μl of the lipofectamine 2000 transfection reagent according to the manufacturers' instructions. 48 hours after transfection the target cells were infected with Phoenix supernatants and selected with 2 μg/ml of puromicin. 293T cells in 6 well plates (60-70% confluency) were transiently transfected for 48 hours with 1 μg of either pcDNA3 (empty vector control), pcDNA3-ANXA2, pcDNA3-ANXA2-Cys-8-Ala, pcDNA3-ANXA2-Cys-8-Ser, pcDNA3-ANXA2-Cys-132-Ala or pcDNA3-ANXA2-Cys-132-Ser plasmids described below using 3 μl of the lipofectamine 2000 transfection reagent according to the manufacturers' instructions.

### Plasmids

pSUPER-retro-ANXA2 shRNA1 was constructed by cloning the dsDNA oligo 5'-GAT CCC CCC TGG TTC AGT GCA TTC AGT TCA AGA GAC TGA ATG CAC TGA ACC AGG TTT TTA-3' and 5'-AGC TTA AAA ACC TGG TTC AGT GCA TTC AGT CTC TTG AAC TGA ATG CAC TGA ACC AGG GGG-3' into pSUPER.retro.puro (OligoEngine), pSUPER-retro-ANXA2 shRNA2 was constructed by cloning the dsDNA oligo 5'-GAT CCC CGT GCA TAT GGG TCT GTC AAT TCA AGA GAT TGA CAG ACC CAT ATG CAC TTT TTA-3' and 5'-AGC TTA AAA AGT GCA TAT GGG TCT GTC AAT CTC TTG AAT TGA CAG ACC CAT ATG CAC GGG-3' into pSUPER.retro.puro (OligoEngine) and the pSUPER-retro-ANXA2 scramble was constructed by cloning the dsDNA oligo 5'-GAT CCC CGT GCA TAT GGG TCT GTC CAT TAG AGA GAT TGA CAG ACC CAT ATG CAC TTT TTA-3' and 5'-AGC TTA AAA AGT GCA TAT GGG TCT GTC AAT CTC TCT AAT GGA CAG ACC CAT ATG CAC GGG-3' into pSUPER.retro.puro (OligoEngine). pSUPER-retro-ANXA2 shRNA3 (Murine) was constructed by cloning the dsDNA oligo into pSUPER.retro.puro (OligoEngine) and pSUPER-retro-ANXA2 shRNA2 (Human specific) was used as ANXA2 scramble (Murine). pcDNA3-ANXA2 plasmid was constructed by PCR amplification of ANXA2 cDNA and cloning it into the pcDNA3 vector. The primers used for PCR amplification were the following: 5'-AAG CTT GGA TCC GCC GCC ACC ATG TCT ACT-3' and 5'-GAT CGC GGC CGC TCA GTC ATC TCC ACC AC-3'. pcDNA3-ANXA2-Cys-8-Ala was constructed by point mutation of the ANXA2 gene in the pcDNA3-ANXA2 vector using the QuikChange II Site-directed mutagenesis kit (Agilent Technologies). The primers used to introduce the point mutation in the ANXA2 gene were the following: 5'-CT GTT CAC GAA ATC CTG GCA AAG CTC AGC TTG GAG GG-3' and 5'-CC CTC CAA GCT GAG CTT TGC CAG GAT TTC GTG AAC AG-3'. pcDNA3-ANXA2-Cys-8-Ser was constructed by point mutation of the ANXA2 gene in the pcDNA3-ANXA2 vector using the QuikChange II Site-directed mutagenesis kit (Agilent Technologies). The primers used to introduce the point mutation in the ANXA2 gene were the following: 5'-GTT CAC GAA ATC CTG AGC AAG CTC AGC TTG GAG GG-3' and 5'-CC CTC CAA GCT GAG CTT GCT CAG GAT TTC GTG AAC-3'. pcDNA3-ANXA2-Cys-132-Ala was constructed by point mutation of the ANXA2 gene in the pcDNA3-ANXA2 vector using the QuikChange II Site-directed mutagenesis kit (Agilent Technologies). The primers used to introduce the point mutation in the ANXA2 gene were the following: 5'-CTC ATT GAG ATC ATC GCT TCC AGA ACC AAC CAG GAG CTG-3' and 5'- CAG CTC CTG GTT GGT TCT GGA AGC GAT GAT CTC AAT GAG-3'. pcDNA3-ANXA2-Cys-132-Ser was constructed by point mutation of the ANXA2 gene in the pcDNA3-ANXA2 vector using the QuikChange II Site-directed mutagenesis kit (Agilent Technologies). The primers used to introduce the point mutation in the ANXA2 gene were the following: 5'-CTC ATT GAG ATC ATC AGC TCC AGA ACC AAC CAG GAG CTG-3' and 5'- CAG CTC CTG GTT GGT TCT GGA GCT GAT GAT CTC AAT GAG-3'.

### Antibodies

The following antibodies were used for western blot analysis: ANXA2 antibody: 610069 (BD Transduction laboratories), S100A10 antibody: 610071 (BD Transduction laboratories), ANXA1 antibody: sc-12740 (Santa Cruz Biotechnology - SCBT), ANXA4 antibody: sc-1930 (SCBT), ANXA5 antibody: sc-8300 (SCBT), ANXA6 antibody: sc-11388 (SCBT), SOD-1 antibody: sc-11407 (SCBT), SOD-2 antibody: sc-30080 (SCBT), PRDX I antibody: sc-7381, PRDX II antibody: sc-33572, PRDX I-IV antibody: sc-33574 (SCBT), catalase antibody: ab1877-10 (AbCam), GPX 1/2 antibody: sc-133160, Trx antibody: sc-20146 (SCBT), phospho-JNK antibody: 9251S (Cell Signaling), JNK antibody: 9252 (Cell Signaling), phospho-p38 antibody: 9211S (Cell Signaling), p38 antibody: 9212 (Cell Signaling), phospho-Akt antibody: 9271S (Cell Signaling), Akt antibody: 9272 (Cell Signaling), actin antibody (AC-40): A3853 (SIGMA), β-tubulin antibody (H-235): sc-9104 (SCBT), JunD antibody: sc-44 (SCBT).

### Immunoprecipitation assays

For Trx, JunD and ANXA2 co-immunoprecipitations, cells were washed two times with PBS, lysed with Triton X-100 lysis buffer (50 mM Tris pH 7.4, 0.15% Triton X-100, 150 mM NaCl, 1.5 mM EGTA, protease inhibitors, 1 mM NaVO_4_, 10 mM NaF- degassed) for 10 minutes on ice. Cell lysates were pre-cleared for 1 h with protein G-Sepharose, incubated with specific antibodies for 1 h and then with 50% slurry of protein G-Sepharose for 1 h. Beads were washed five times with 500 μl of lysis buffer, resuspended with 25 μl 2X SDS-PAGE loading buffer, boiled for 5 minutes, subjected to SDS-PAGE and analysed by western blotting. The following antibodies were used for immunoprecipitation studies: ANXA2: D1/274.5 mouse monoclonal (made in house), JunD: sc-44 (SCBT) and Trx: sc-20146 (SCBT).

### Western blot analysis

For western blot analysis 20 μg of cell lysates, unless noted, were subjected to SDS-PAGE, transferred onto a nitrocellulose membrane, incubated with appropriate antibodies and visualized using a Licor Odyssey scanner (Li-cor Biosciences). Quantification of protein bands was done using the Licor Odyssey scanner software.

### Determination of redox sensitive cysteine oxidation

Redox sensitive cysteine oxidation was determined using the thiol-specific biotinylation reagent, BIAM (SIGMA). For experiments that utilized cultured cells, after mock, EGF or H_2_O_2_ treatment, cells were washed two times with PBS and scraped with BIAM lysis buffer (10 μM BIAM, 50 mM Tris pH 7.4, 0.2% Triton X-100, 200 mM NaCl, 2 mM EGTA, 1 mM EDTA, 5 μg/ml catalase, protease inhibitors, 1 mM NaVO_4_, 10 mM NaF-degassed). The resultant lysates were incubated for 20 minutes at 37^o^C, after which 2 mM iodoacetamide (IA) was added to block any further protein labeling. In other experiments, lysates were prepared by homogenization of tissues or tumor samples with lysis buffer (50 mM Tris pH 7.4, 0.2% Triton X-100, 200 mM NaCl, 2 mM EGTA, 1 mM EDTA, protease inhibitors, 1 mM NaVO_4_, 10 mM NaF-degassed) followed by incubation at 4°C for 15 minutes and centrifugation at 20000 g for 20 minutes at 4°C. The lysates were incubated with 20 μM BIAM for 20 minutes at 37^o^C followed by addition of 2 mM IA. In order to isolate BIAM-labeled proteins, cell extracts that had been labeled with BIAM were incubated with 50 μl of streptavidin Dynabeads (Invitrogen) for 1 hour at 4^o^C with rotation. Streptavidin beads were washed 3 times with 500 μl of lysis buffer, resuspended with 25 μl of 2X SDS-PAGE loading buffer and subjected to SDS-PAGE followed by western blotting. All human samples were obtained with patients' informed consent and the work was approved by the Capital Health Research Ethics Board.

### NADPH assay

250 μg of TIME cell lysates were either non-treated or treated with H_2_O_2_ for 15 minutes. Lysates were incubated with 100 μg/ml of catalase for 10 minutes at 37^o^C, after which 500 μM of NADPH was added to the samples and incubated for 30 minutes at 37^o^C. Samples were incubated with 10μM BIAM reagent and labeled proteins were purified by incubation with streptavidin beads. Cell extracts and BIAM labeled purified samples were subjected to SDS-PAGE and analyzed by western blotting.

### Trx assay

2.5 μM of either ANXA2 or ANXA1 were either non treated or treated with 100 μM of H_2_O_2_ in the presence or absence of 20 μM Trx for 15 minutes at 37°C. Samples were labeled with 20 μM BIAM for 20 minutes at 37^o^C, subjected to SDS-PAGE under non reducing conditions and analyzed by western blotting.

### Intracellular ROS measurement

The fluorescent dye DCF-DA (Invitrogen) was used to measure the levels of ROS in cells. 2 X 10^4^ cells/well were seeded in 96 wells plate and incubated overnight at 37^o^C. Cell medium was removed and replaced with 1% FBS medium containing 50 μM DCF-DA. Cells were incubated for 30 minutes at 37^o^C, washed with PBS and complete medium was added. Cells were treated with H_2_O_2_ ± NAC, incubated for 1 hour at 37^o^C and fluorescence was measured (Excitation: 488 nm, Emission: 535 nm) using a fluorometer plate reader.

### Cell viability assay

Cell viability was determined by using the CellTiter 96® AQueous Non-Radioactive Cell Proliferation Assay (Promega) according to the manufacturer's instructions.

### Tumor development in NOD-SCID mice

NOD-SCID mice were injected subcutaneously into the right flank with 2 X 10^6^ HT1080 ANXA2 shRNA 2, HT1080 ANXA2 scramble, A549 ANXA2 shRNA2 or A549 ANXA2 scramble cells in 100 μl of PBS in the absence or presence of 150 mg/kg of NAC administered intraperitoneally every two days. Tumor volume was measured every two/three days by caliper measurement and volume calculated by the formula (V=0.5 X L X W^2^). At the experimental endpoint mice were euthanized by CO_2_ inhalation, the ANXA2 depleted HT1080 and control tumors were removed and protein lysates were prepared. Tumors were homogenized with 1ml lysis buffer (50 mM Tris pH 7.4, 0.2% Triton-X100, 200 mM NaCl, 1 mM EDTA, 2 mM EGTA, 1 mM NaVO_4_, 10 mM NaF and protease inhibitors-degassed). Homogenates were centrifuged at 20000 g for 20 minutes at 4°C and supernatants were stored. All mouse experiments were performed in accordance with protocols approved by the University Committee on Laboratory Animals (UCLA) at Dalhousie University, Halifax, N.S., Canada.

### Statistical analysis

The statistical significance of the difference in cytotoxicity, DCF quantification and protein oxidation was evaluated using two tailed Student's t test. The statistical difference in tumor growth between the ANXA2 depleted HT1080 versus control cells with or without NAC and ANXA2 depleted A549 versus control cells was analyzed using the 2-way Analysis of Variance (ANOVA) test. These data analyses were performed using the Prism software (GraphPad, San Diego, CA). In every case a P value of less than 0.05 (*), less than 0.01(**) and 0.001 (***) was considered statistically significant.

### Inventory of Supplemental data

Supplemental data includes supplemental methods, five figures and one table.

## Supplementary Data, Figures and Tables






