# Supplementary material for: Annexin A2 is a novel Cellular Redox Regulatory Protein involved in Tumorigenesis
Source: Oncotarget. 2011 Dec 20;2(12):1075–93. doi: 10.18632/oncotarget.375 (PMC3282068; doi:10.18632/oncotarget.375)
Supplement: Supplementary file 1 [file oncotarget-02-1075-s001.pdf]

SUPPLEMENTARY DATA

SUPPLEMENTARY EXPERIMENTAL PROCEDURES

Cellular growth rate measurement

2 X 10^4 HT1080 ANXA2 shRNA2, HT1080 ANXA2 scramble, A549 ANXA2 shRNA2 or A549 scramble cells were plated in each well of 12 wells plates. Cells from 3 wells (N=3) were counted for each time point: 24, 48, 72, 96 and 144 hours for HT1080 cells and 24, 48, 72, 96, 144, 168 and 192 hours for A549 cells using a haemocytometer.

NEM-biotin labeling assay

Cells were washed two times with PBS and scraped with NEM lysis buffer (10 µM NEM, 50 mM Tris pH 7.4, 0.2% Triton X-100, 200 mM NaCl, 2 mM EGTA, 1 mM EDTA, protease inhibitors, 1 mM $NaVO_4$, 10 mM NaF-degassed). The resultant lysates were incubated for 20 minutes at 37°C, after what the lysates were centrifuged at 20000 g for 15 minutes. 20 µg of each labeled cell lysate was subjected to SDS-PAGE followed by western blot analysis.
