# Supplementary material for: Annexin A2 is a novel Cellular Redox Regulatory Protein involved in Tumorigenesis
Source: Oncotarget. 2011 Dec 20;2(12):1075–93. doi: 10.18632/oncotarget.375 (PMC3282068; doi:10.18632/oncotarget.375)
Supplement: Supplementary file 2 [file oncotarget-02-1075-s002.pdf]

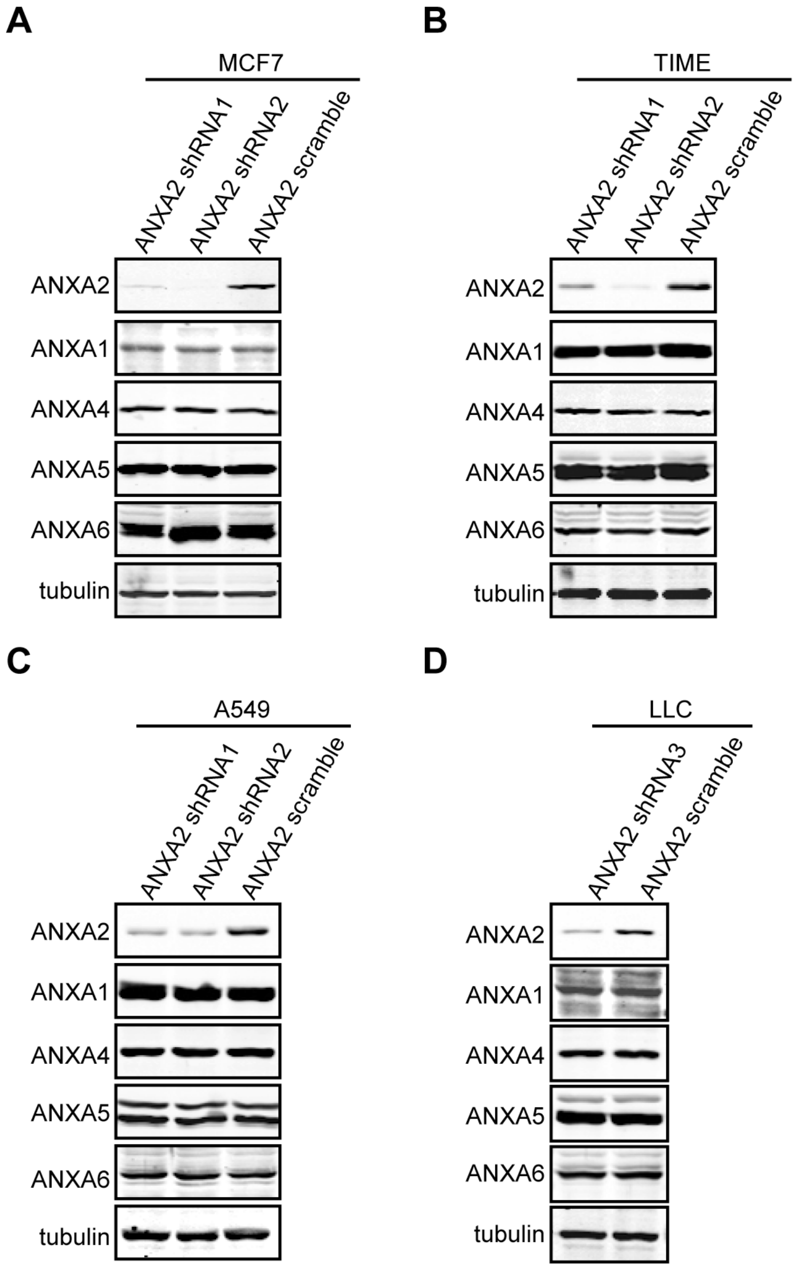

Figure S1: Analysis of expression of annexins in ANXA2 knockdown cell lines. (A) MCF7; (B) TIME and (C) A549 cells were infected with retroviral supernatants from Phoenix cells transfected with pSUPER-retro-ANXA2 shRNA1, pSUPER-retro-ANXA2 shRNA2 or pSUPER-retro-ANXA2 scramble as indicated. (D) LLC cells were infected with retroviral supernatants from Phoenix cells transfected with pSUPER-retro-ANXA2 shRNA3 (Murine) or pSUPER-Retro-ANXA2 scramble (Murine) as indicated. 20 µg of each cell lysate was subjected to SDS-PAGE followed by western blot analysis with the antibodies indicated.

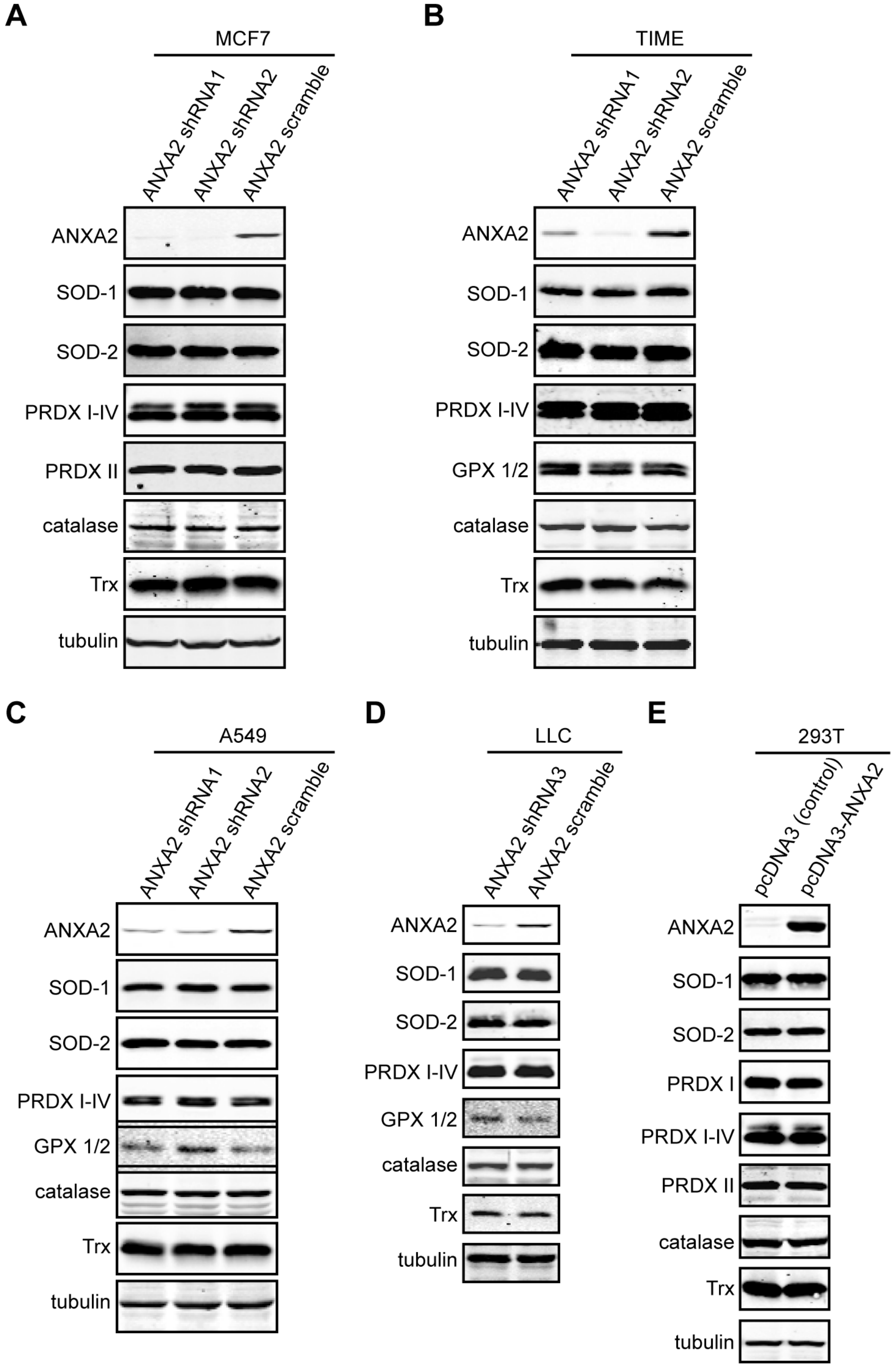

Figure S2: ANXA2 expression levels do not alter the levels of redox proteins. 20 μg of each ANXA2 knockdown or control (scramble) cell lysates: (A) MCF7, (B) TIME, (C) A549 or (D) LLC were subjected to SDS-PAGE followed by western blot analysis with the antibodies indicated. Note: We were not able to detect glutathione peroxidase 1/2 (GPX 1/2) expression in MCF7 cells or peroxiredoxin II (PRDX II) expression in TIME, A549 or LLC cells by western blot analysis. (E) 293T cells were transiently transfected with pcDNA3 (lane 1) or pcDNA3-ANXA2 (lane 2) for 72 h. 20 μg of each cell lysate were subjected to SDS-PAGE followed by western blotting with the antibodies indicated. Note: We were not able to detect glutathione peroxidase 1/2 (GPX 1/2) expression in 293T cells.

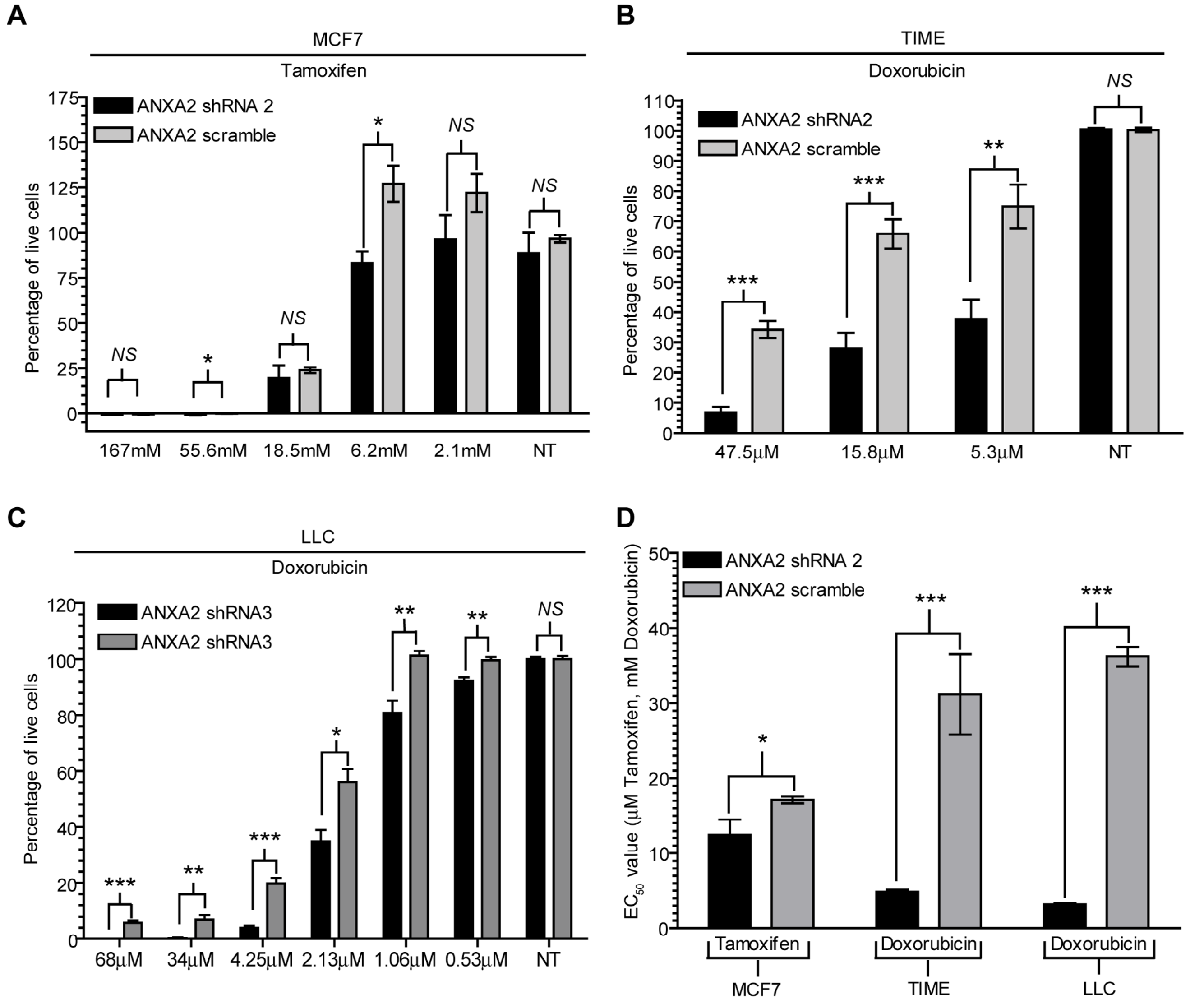

Figure S3: ANXA2 depleted cells are more sensitive to chemotherapeutic drugs induced death. (A) MCF7 ANXA2 shRNA2 or MCF7 ANXA2 scramble cells were treated with different concentrations of tamoxifen for 48 hours. (B) TIME ANXA2 shRNA2 or TIME ANXA2 scramble cells and (C) LLC ANXA2 shRNA 3 or LLC ANXA2 scramble cells were treated with different concentrations of doxorubicin for 48 hours. Cell viability was determined using the CellTiter 96® AQueous Non-Radioactive Cell Proliferation Assay (Promega) according to the manufacturer's instructions. Average 490 nm absorbance for NT cells was set as 100% viability (D) Ec_{50} graphs for the cells treated with either tamoxifen or doxorubicin as indicated.

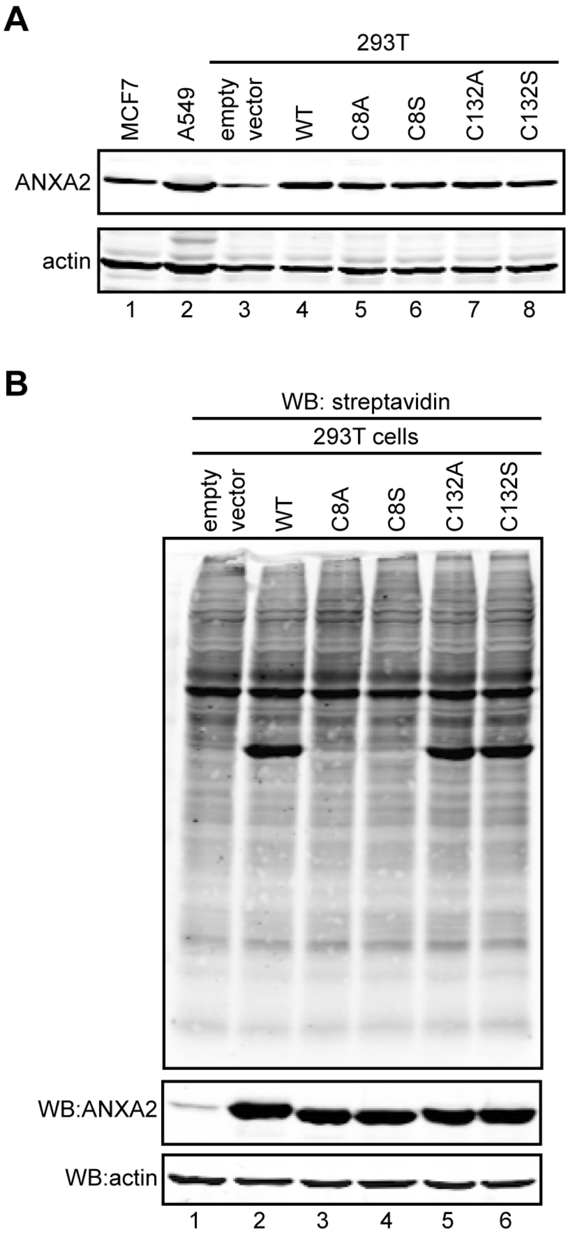

Figure S4: NEM labeling of 293T cells expressing ANXA2 WT and cysteine mutants. (A) 293T cells were transiently transfected with pcDNA3 empty vector (lane 3), pcDNA3-ANXA2 (lane 4), pcDNA3-ANXA2-Cys-8-Ala (lane 5), pcDNA3-ANXA2-Cys-8-Ser (lane 6), pcDNA3-ANXA2-Cys-132-Ala (lane 7) or pcDNA3-ANXA2-Cys-132-Ser (lane 8) for 48 hours. 20 μg of each cell lysate, MCF7 cell lysate (lane 1) or A549 cell lysate (lane 2) was subjected to SDS-PAGE, followed by western blotting with the antibodies indicated. (B) 293T cells were transiently transfected with pcDNA3 (lane 1), pcDNA3-ANXA2 (lane 2), pcDNA3-ANXA2-Cys-8-Ala (lane 3), pcDNA3-ANXA2-Cys-8-Ser (lane 4), pcDNA3-ANXA2-Cys-132-Ala (lane 5) or pcDNA3-ANXA2-Cys-132-Ser (lane 6) plasmids for 48 hours. 20 μg of each cell lysate was labeled with 20 μM NEM, subjected to SDS-PAGE followed by western blotting with the antibodies indicated.

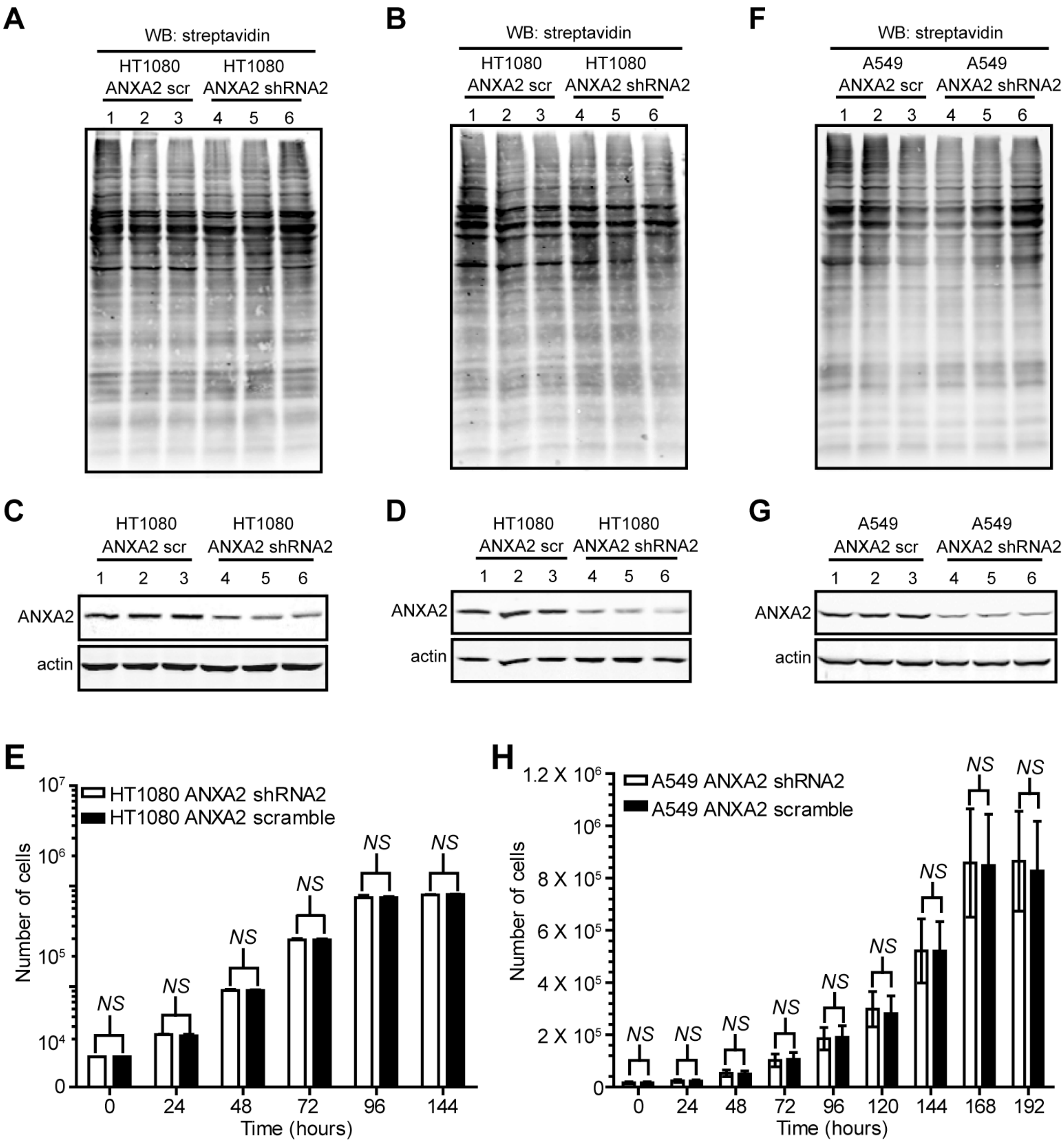

Figure S5: Analysis of the protein redox status and growth rate of HT1080 ANXA2 shRNA2, HT1080 ANXA2 scramble, A549 ANXA2 shRNA2 and A549 scramble cells. (A) HT1080 ANXA2 scramble cells (lanes 1-3) and HT1080 ANXA2 shRNA2 cells (lanes 4-6) selected with puromicin for 1 week or (B) selected with puromicin for 3 weeks were labeled with 20 µM BIAM, subjected to SDS-PAGE followed by western blot analysis with a streptavidin probe. (C) 20 µg of protein lysates from HT1080 ANXA2 scramble cells (lanes 1-3) and HT1080 ANXA2 shRNA2 cells (lanes 4-6) selected for 1 week with puromicin or (D) selected for 3 weeks with puromicin were subjected to SDS-PAGE followed by western blot analysis with the antibodies indicated. (E) 2 X 10^4 HT1080 ANXA2 shRNA2 or HT1080 ANXA2 scramble cells were plated in each well of 12 wells plates. Cells from 3 wells (N=3) were counted for each time point: 24, 48, 72, 96 and 144 hours. Results are represented as ± StDev. (F) A549 ANXA2 scramble cells (lanes 1-3) and A549 ANXA2 shRNA2 cells (lanes 4-6) selected with puromicin for 1 week were labeled with 20 µM BIAM, subjected to SDS-PAGE followed by western blot analysis with a streptavidin probe. (G) 20 µg of protein lysates from A549 ANXA2 scramble cells (lanes 1-3) and A549 ANXA2 shRNA2 cells (lanes 4-6) selected for 1 week with puromicin were subjected to SDS-PAGE followed by western blot analysis with the antibodies indicated. (H) 2 X 10^4 A549 ANXA2 shRNA2 or A549 ANXA2 scramble cells were plated in each well of 12 wells plates. Cells from 3 wells (N=3) were counted for each time point: 24, 48, 72, 96, 144, 168 and 192 hours. Results are represented as ± StDev.
