# Supplementary material for: Annexin A2 is a novel Cellular Redox Regulatory Protein involved in Tumorigenesis
Source: Oncotarget. 2011 Dec 20;2(12):1075–93. doi: 10.18632/oncotarget.375 (PMC3282068; doi:10.18632/oncotarget.375)
Supplement: Supplementary file 3 [file oncotarget-02-1075-s003.pdf]

SUPPLEMENTARY TABLES

Table S1

Ec_{50} values for ANXA2 KD and control cells treated with H_2O_2 and chemotherapeutic agents.

Cell line	H_2O_2 treatment (Ec_{50})	Standard deviation (StDev)
MCF7 p36 shRNA2	1.07 mM	± 0.056 mM
MCF7 p36 scramble	4.377 mM	± 0.707 mM
TIME p36 shRNA2	0.462 mM	± 0.007 mM
TIME p36 scramble	0.682 mM	± 0.008 mM
LLC p36 shRNA3	0.797 mM	± 0.178 mM
LLC p36 scramble	6.605 mM	± 0.876 mM
**Cell line**	**Etoposide treatment (Ec_{50})**	**Standard deviation (StDev)**
MCF7 p36 shRNA2	0.189 µM	± 0.019 µM
MCF7 p36 scramble	0.645 µM	± 0.296 µM
TIME p36 shRNA2	0.0163 µM	± 0.005 µM
TIME p36 scramble	0.133 µM	± 0.053 µM
LLC p36 shRNA3	0.01 µM	± 0.0015 µM
LLC p36 scramble	0.25 µM	± 0.12 µM
**Cell line**	**Tamoxifen treatment (Ec_{50})**	**Standard deviation (StDev)**
MCF7 p36 shRNA2	12.5 µM	± 3 µM
MCF7 p36 scramble	17.1 µM	± 1 µM
**Cell line**	**Doxorubicin treatment (Ec_{50})**	**Standard deviation (StDev)**
TIME p36 shRNA2	4.85 µM	± 0.5 µM
TIME p36 scramble	31.2 µM	± 10.6 µM
LLC p36 shRNA3	3.1 µM	± 0.53 µM
LLC p36 scramble	36.2 µM	± 2.6 µM
